# CRISPR/Cas9-mediated *CHS2* mutation provides a new insight into resveratrol biosynthesis by causing a metabolic pathway shift from flavonoids to stilbenoids in *Vitis davidii* cells

**DOI:** 10.1093/hr/uhae268

**Published:** 2024-10-09

**Authors:** Gongti Lai, Peining Fu, Liyuan He, Jianmei Che, Qi Wang, Pufu Lai, Jiang Lu, Chengchun Lai

**Affiliations:** Institute of Food Science and Technology, Fujian Academy of Agricultural Sciences, No. 247 Wusi Road, Gulou District, Fuzhou 350003, China; Key Laboratory of Subtropical Characteristic Fruits, Vegetables and Edible Fungi Processing (Co-construction by Ministry and Province), Ministry of Agriculture and Rural Affairs, No. 247 Wusi Road, Gulou District, Fuzhou 350003, China; Center for Viticulture and Enology, School of Agriculture and Biology, Shanghai Jiao Tong University, No. 800 Dongchuan Road, Minhang District, Shanghai 200240, China; Institute of Food Science and Technology, Fujian Academy of Agricultural Sciences, No. 247 Wusi Road, Gulou District, Fuzhou 350003, China; Institute of Resources, Environment and Soil Fertilizer, Fujian Academy of Agricultural Sciences, No. 247 Wusi Road, Gulou District, Fuzhou 350003, China; Institute of Food Science and Technology, Fujian Academy of Agricultural Sciences, No. 247 Wusi Road, Gulou District, Fuzhou 350003, China; Key Laboratory of Subtropical Characteristic Fruits, Vegetables and Edible Fungi Processing (Co-construction by Ministry and Province), Ministry of Agriculture and Rural Affairs, No. 247 Wusi Road, Gulou District, Fuzhou 350003, China; Institute of Food Science and Technology, Fujian Academy of Agricultural Sciences, No. 247 Wusi Road, Gulou District, Fuzhou 350003, China; Key Laboratory of Subtropical Characteristic Fruits, Vegetables and Edible Fungi Processing (Co-construction by Ministry and Province), Ministry of Agriculture and Rural Affairs, No. 247 Wusi Road, Gulou District, Fuzhou 350003, China; Center for Viticulture and Enology, School of Agriculture and Biology, Shanghai Jiao Tong University, No. 800 Dongchuan Road, Minhang District, Shanghai 200240, China; Institute of Food Science and Technology, Fujian Academy of Agricultural Sciences, No. 247 Wusi Road, Gulou District, Fuzhou 350003, China; Key Laboratory of Subtropical Characteristic Fruits, Vegetables and Edible Fungi Processing (Co-construction by Ministry and Province), Ministry of Agriculture and Rural Affairs, No. 247 Wusi Road, Gulou District, Fuzhou 350003, China

## Abstract

Resveratrol is an important phytoalexin that adapts to and responds to stressful conditions and plays various roles in health and medical therapies. However, it is only found in a limited number of plant species in low concentrations, which hinders its development and utilization. Chalcone synthase (CHS) and stilbene synthase (STS) catalyze the same substrates to produce flavonoids and resveratrol, respectively. However, it remains unclear how CHS and STS compete in metabolite synthesis. In this study, two *CHS2* mutant cell lines (MT1 and MT2) were generated using CRISPR/Cas9 genome editing. These *CHS2* mutant cell lines exhibited abundant mutations in *CHS2*, leading to the premature termination of protein translation and subsequent *CHS2* knockout. Amplicon sequencing confirmed comprehensive *CHS2* knockout in MT1, whereas the wild-type sequence remained predominant in the MT2 cell line. Transcriptome and RT-qPCR results showed a significant downregulation of genes involved in flavonoid biosynthesis, including *CHS2*, *CHS3*, *F3H*, *F3’H*, *DFR*, *FLS*, *LDOX*, among others, resulting in decreased flavonoid accumulation, such as anthocyanins, proanthocyanidins, quercetin, and kaempferol. Conversely, *STS* genes involved in stilbenoid biosynthesis were upregulated competing with the flavonoid pathway. Consequently, there was a marked increase in stilbenoids, including resveratrol, piceatannol, piceid, and pterostilbene, with a 4.1-fold increase in resveratrol and a 5.3-fold increase in piceid (a derivative of resveratrol) observed in *CHS2* mutant cell lines. This research demonstrates that *CHS2* mutation induces a shift from flavonoid biosynthesis towards stilbenoid biosynthesis, offering new insights into metabolite biosynthesis and regulation, as well as an alternative solution for natural resveratrol production, and a novel breeding approach for eliminating non-target agronomic traits using CRISPR-Cas9.

## Introduction

Resveratrol, a stilbenoid, is an important natural polyphenol derived from phenylpropanoid biosynthesis [1]. Resveratrol and its derivatives (stilbenoids) play crucial biological roles in stress response by ameliorating ultraviolet (UV) radiation damage, enhancing resistance to pathogen infection, and serving as signal molecules for plants–environment interactions [2–5]. Resveratrol is well-documented for its wide range of physiological and pharmacological health benefits, including antioxidant, anti-inflammatory, anti-aging, anti-proliferative, and anti-diabetic effects [6–10]. Nevertheless, resveratrol is present in only a limited number of plants [11, 12], such as giant knotweeds [13], grapes [14], and peanuts [15]. The commercial development and application of natural resveratrol face challenges due to limited resources, low concentrations, complex extraction procedures, and purification difficulties [16]. Consequently, alternative solutions for resveratrol production have become a major research focus.

Chemical synthesis is an effective approach for large-scale resveratrol production in industrial applications. However, it often results in unwanted compound contamination, leading to purity issues [16] and safety concerns [17]. Microbial fermentation, utilizing synthetic biology strategies, has shown significant progress in resveratrol production, emerging as a promising method for cost-effective production [18]. Commonly used microbial hosts include *Escherichia coli* [19], *Corynebacterium glutamicum* [20], *Streptomyces venezuelae* [21], and yeasts such as *Saccharomyces cerevisiae* [22, 23] and *Yarrowia lipolytica* [24, 25]. Despite its potential, microbial fermentation faces challenges including byproduct enrichment and unwanted metabolite contamination. Additionally, there is a significant difference in bioactivity between heterologous expression products and plant-derived natural products, possibly due to a lack of abundant post-translational modifications and complex product processing mechanisms. Furthermore, protein misfolding and insoluble inclusion bodies are also common when expressing exogenous genes in microbes [26]. These issues underscore the need for alternative approaches to resveratrol production and utilization, complementing direct plant extraction, chemical synthesis, and microbial fermentation.

Modern biotechnological applications, such as cell engineering and genetic manipulation, offer new strategies for producing target natural products *in vitro* [27]. Plant cell factory-based synthetic biology provides a safe, cost-effective, ecological, and efficient approach for resveratrol production, with numerous studies exploring this method. The overexpression of the stilbene synthase (*STS)* gene, a key component in resveratrol synthesis, combined with biotic and abiotic treatments such as *Uncinula necator*, ultraviolet-C (UV-C), external metabolic precursor feeding, and other elicitor inductions, offers efficient strategies [28–30]. Enhanced resveratrol accumulation through *STS* overexpression demonstrates forward manipulation of target metabolite regulation. Given that both resveratrol and flavonoids are derived from phenylpropanoid biosynthesis, STS, and chalcone synthase (CHS) catalyze the same substrates (one molecule of p-coumaroyl-CoA and three molecules of malonyl-CoA) to produce stilbenoids and flavonoids, respectively [31]. Considering the competitive relationship between *STS* and *CHS* [32, 33], it is hypothesized that *CHS* mutation or knockout could shift the metabolic pathway from flavonoid to stilbenoid synthesis, thereby promoting resveratrol biosynthesis, representing a reverse manipulation of target metabolite regulation.

In this study, the previously isolated *VdCHS2* gene, which was significantly up-regulated in *V. davidii* during culture, was chosen as the target gene. Gene editing of *VdCHS2* was performed using the CRISPR (Clustered Regularly Interspaced Short Palindromic Repeats)/Cas9 system, a robust, convenient, and highly efficient gene editing/knockout tool [34]. *CHS2* mutant cell lines were constructed and screened for high-yield resveratrol production. Gene expression and metabolite accumulation were analyzed for flavonoid and stilbenoid biosynthesis. This study aims to address the high cost, lengthy production cycle, and low extraction efficiency of natural resveratrol production, providing a theoretical and practical foundation for the exploration and utilization of valuable gene resources and offering new insights into the regulation of metabolite regulation.

## Results

### CRISPR/Cas9 vector construction and mutant cell line selection

**Figure 1:**
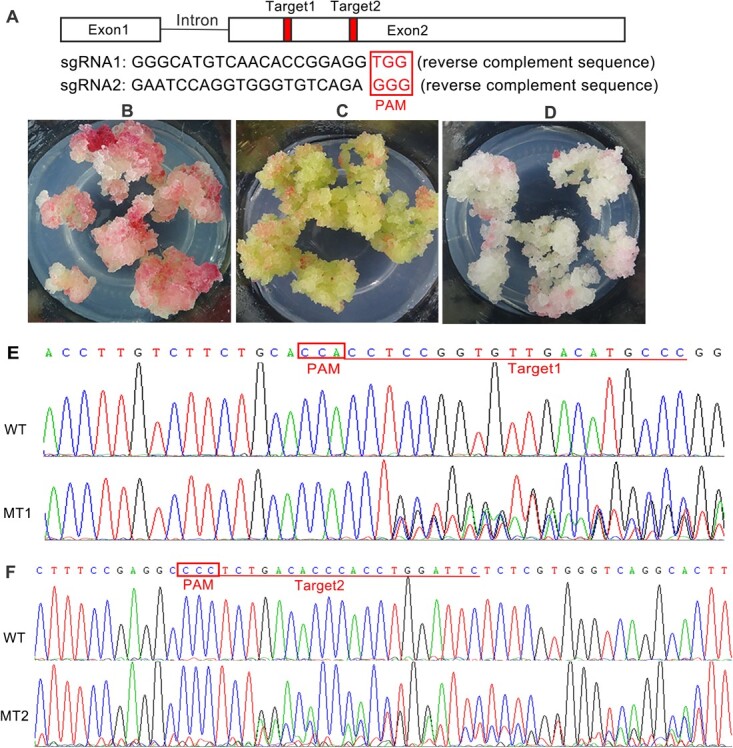
Phenotypes and sequencing chromatograms of *CHS2* in mutant cell lines. (A) *CHS2* gene structure and target site selection for CRISPR/Cas9 editing. Two single target sites (target1 and target2) were chosen in exon2 of *CHS2* sequence. (B) Wild-type cell line, (C) MT1 and (D) MT2 mutant cell lines of *V. davidii*. MT1 and MT2 correspond to mutations at target1 and target2 of *CHS2*, respectively. (E, F) Sequencing chromatograms of target1 and target2 in MT1 and MT2 derived from PCR products, showing overlapping traces indicative of mutations.

A previously isolated gene, *VdCHS2* was selected as the target gene, which was significantly upregulated in *V. davidii* during culture. Two single target sites (target1 and target2) within the second exon of *CHS2* at ORF_395–414_ and ORF_604–623_ were selected for CRISPR/Cas9 vector construction (Fig. 1A). Two single *CHS2* target CRISPR/Cas9 recombinant plasmids were introduced into *Agrobacterium tumefaciens* (GV3101) and transformed to generate *CHS2* mutations in *V. davidii* cells. Cell lines were selected based on hygromycin (Hyg) resistance, and MT1 and MT2 were preliminarily identified as mutant cell lines. In the early culture phase, MT1 and MT2 cell lines exhibited green and white-yellow calli, respectively. After 25 d of culture, a light red phenotype emerged on the callus surface, whereas the WT calli turned red (Fig. 1B–D). This phenotypic variation suggested that anthocyanin biosynthesis was suppressed in *CHS2* mutant cell lines. Direct sequencing of PCR products revealed overlapping traces around the target sites in the sequencing chromatograms of MT1 (Fig. 1E) and MT2 (Fig. 1F) samples, confirming mutations. A putative off-target for target1 was predicted at *CHS3*, exhibiting only two nucleotide variations (Fig. S1A). PCR products of the putative off-target were obtained (Fig. S1B), and sequencing results confirmed absence of mutations (Fig. S1C), indicating no off-target effects of target1 in *CHS3*.

### Multiple mutation types leading to *CHS2* gene knockout

**Figure 2:**
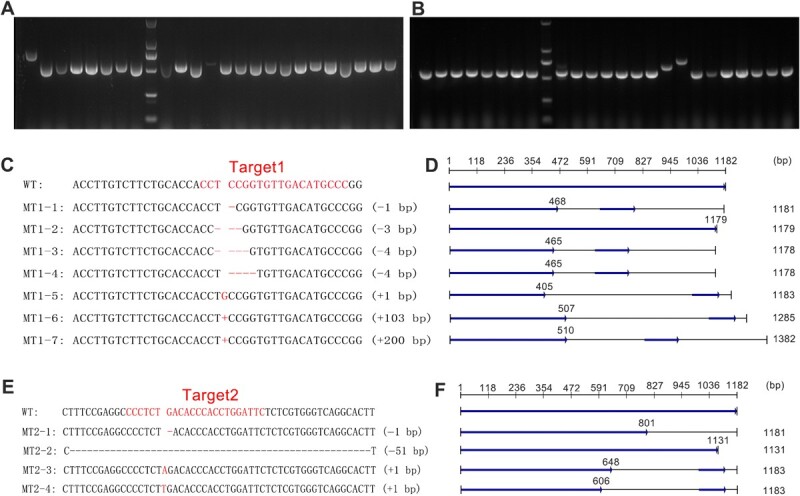
Gel electrophoretogram and mutation characteristics of *CHS2* sequences. (A, B) Gel electrophoretogram of colony PCR to identify target mutations in MT1 and MT2. (C, E) Mutation characteristics of the *CHS2* gene in MT1 and MT2, showing seven and four types of sequence mutations in MT1 and MT2, respectively. (D, F) Protein translation characteristics encoded by *CHS2* mutation sequences in MT1 and MT2. Most mutations result in premature termination of protein translation, causing *CHS2* gene knockout.

The sequencing chromatograms were subsequently decoded using the DSD method, which proved ineffective due to the complexity of the mutations. The previous PCR products were further subcloned, and the electrophoretic migration of clone-based PCR revealed bands of varying sizes (Fig. 2A and B), indicating diverse mutation types in MT1 and MT2. Sequencing of random clones identified seven mutation types in MT1 (Fig. 2C), including four deletions (C, TCC, TCCG, and CCGG deletion) and three insertions (G, 103 bp, and 200 bp insertions). Among these, 85.7% resulted in premature termination of protein translation at ORF_405–510_ (Fig. 2D), leading to *CHS2* knockout, with only the TCC deletion resulting in a complete protein translation albeit with one codon deletion. MT2 displayed four mutation types (Fig. 2E), including two deletions (G and 51 bp deletion) and two insertions (A and T insertions), with 75% leading to premature termination of protein translation at ORF_606–801_ (Fig. 2F). The mutation type of 51 bp deletion also resulted in a CHS2 protein that was 17 amino acids shorter. CRISPR/Cas9 based genome editing thus introduced abundant mutations in *CHS2*, causing premature termination of protein translation and resulting in *CHS2* knockout. MT1 exhibited a higher frequency of *CHS2* knockout and shorter normal translation sequences.

### Amplicon sequencing revealed high-throughput screening of *CHS2* mutation

**Figure 3:**
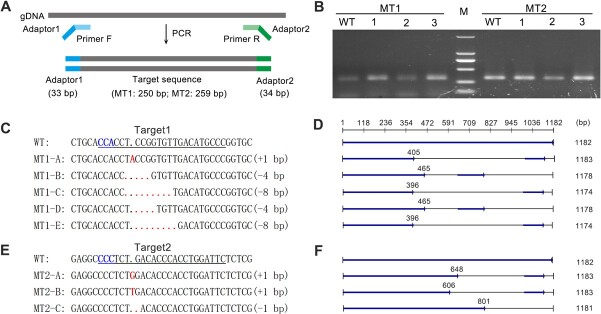
PCR amplification for amplicon-seq and sequence mutation information. (A) Schematic diagram of PCR and (B) gel electropherogram for amplicon sequencing. (C, E) The dominant mutation types of *CHS2* genes in MT1 and MT2. (D, F) Protein translation characteristics encoded by *CHS2* mutation sequence in MT1 and MT2. Underlines indicate the gene editing targets.

Cloning and sequencing of PCR-derived products revealed various mutations in *CHS2*, but the limited number of sequenced clones did not capture the complete mutation spectrum. To further screen for mutations, amplicon sequencing was conducted, for which specific PCR primers with adaptors were designed (Fig. 3A) and gel electropherogram confirmed the amplification of target products (Fig. 3B). The amplicon-seq data have been deposited in the Genome Sequence Archive (GAS) database (Accession No. CRA017913), and the data quality assessment was listed in Table S3. MT1 samples exhibited 7–9 types of mutations (Table S4), including one insertion (A) and four deletions (TCCG, TCCGGTGT, CCGG, CCGGTGTT) at positions 397–405 bp (Table 1, Fig. 3C). The MT2 cell line showed 10 to 14 types of mutations (Table S5), with two insertions (G, T) and one deletion (G) at positions 606–607 bp (Table 1, Fig. 3E). Notably, 99.87% and 46.67% of the mutations in MT1 and MT2, respectively, resulted in premature termination of protein translation, respectively, leading to *CHS2* knockout (Fig. 3D, F). These foundings suggested that *CHS2 is* comprehensively knocked out in MT1, whereas the wild-type sequence remains the predominant genotype in the MT2 cell line.

**Table 1:** Mutation information of *CHS2* targets

Cell line	Mutation	Type	Position	Sequence	Sequence count				Ratio (%)
					Sample 1	Sample 2	Sample 3	Average	
MT1	WT	-	-	-	8	2	9	6	0.13
	MT1-A	+1	397	A	2760	3687	3417	3288	63.18
	MT1-B	−4	397–400	TCCG	1447	1917	1554	1639	31.54
	MT1-C	−8	397–404	TCCGGTGT	167	75	168	137	2.73
	MT1-D	−4	398–401	CCGG	69	48	81	66	1.30
	MT1-E	−8	398–405	CCGGTGTT	48	48	66	54	1.05
	MT2-Others	-	-	-	5	5	3	4	0.08
MT2	WT	-	-	-	3202	3031	2799	3011	53.33
	MT2-A	+1	606	G	1716	1575	1513	1601	28.36
	MT2-B	+1	606	T	508	525	440	491	8.70
	MT2-C	−1	607	G	483	545	445	491	8.70
	MT2-Others	-	-	-	70	72	13	52	0.92

### DAM (differentially accumulated metabolite) analysis revealed that flavanoid biosynthesis was suppressed and stilbenoid biosynthesis was enhanced in *CHS2* mutant cell lines

**Figure 4:**
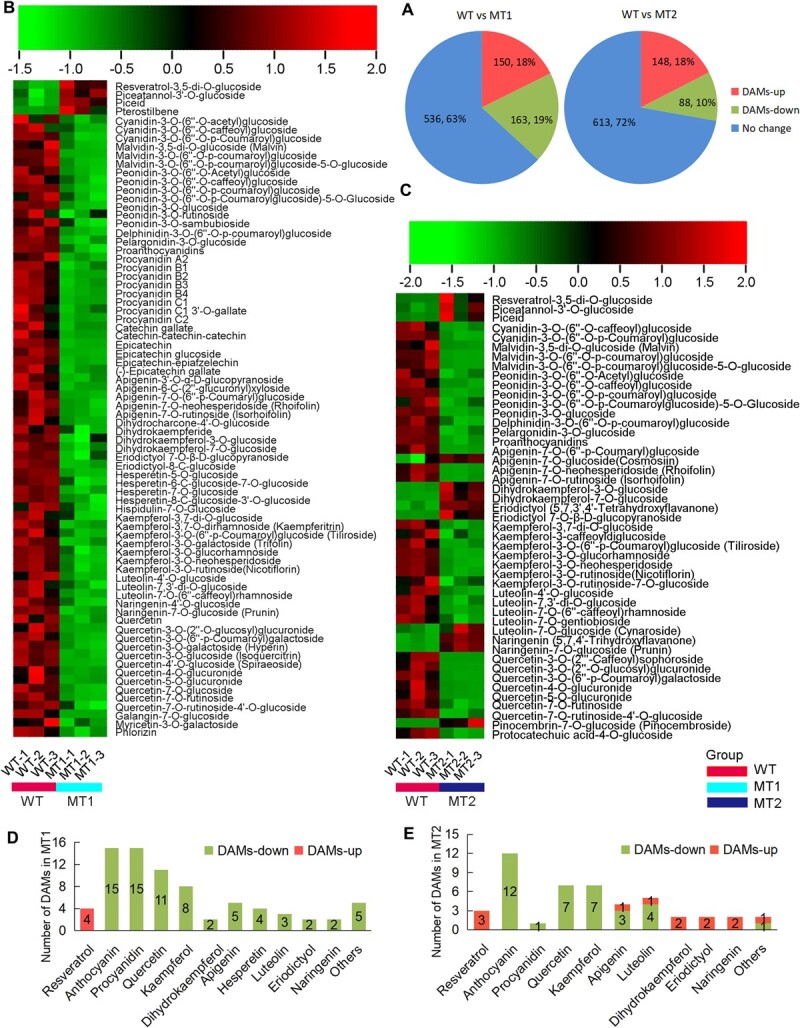
Differential accumulation metabolites (DAMs) in *CHS2* mutant cell lines. (A) DAMs analysis of MT1 and MT2. A otal of 850 metabolites were detected in mutant cell lines, with 313 and 236 DAMs identified in MT1 and MT2, respectively. Clustering heat map of DAMs in (B) MT1 and (C) MT2 involved in stilbenoid and flavonoid pathway. (D, E) Number of DAMs in MT1 and MT2, with the X-axis representing metabolites and their derivatives.

To further explore the accumulation of flavonoids and stilbenoids following *CHS2* gene editing, nontargeted metabolomic analysis was performed on the wild type (WT) *V. davidii* callus cell line and *CHS2* mutant callus cells (MT1 and MT2) at 25 d. The metabolomics data for all identified metabolites and differentially accumulated metabolite (DAMs) were deposited in OMIX database (Accession No. OMIX007203). A clustering heat map of total metabolite accumulation and sample correlation is shown in Figs S2 and S3. DAM analysis identified a total of 313 (150 upregulated and 163 downregulated) and 236 (148 upregulated and 88 downregulated) DAMs in MT1 and MT2, respectively, using variable importance in the projection (VIP) > 1 and FC (Fold change) >2 (Fig. 4A). Among these, four stilbenoids and 72 flavonoids in MT1 (Table S6), and three stilbenoids and 44 flavonoids in MT2 (Table S7) were identified as significant DAMs.

The DAM clustering heat map indicated significant upregulation of four and three differentially accumulated stilbenoids (resveratrol derivatives) in MT1 and MT2, respectively, including resveratrol-3-5-di-O-glucoside, piceatannol-3’-O-glucoside, piceid, and pterostilbene (Fig. 4B and C). Conversely, all flavonoids in MT1 and most in MT2 were significantly downregulated, including anthocyanins, proanthocyanidins, quercetin, kaempferol, dihydrokaempferol, apigenin, hesperetin, luteolin, eriodictyol, naringenin, and their derivatives. Among these, anthocyanins, proanthocyanidins, quercetin, kaempferol, and their derivatives constituted the majority of the DAMs (Fig. 4D and E). These findings demonstrate that flavonoid biosynthesis was suppressed, whereas stilbenoid biosynthesis was enhanced.

### *CHS2* mutations led to significant increases in stilbenoids but decreases in flavonoids

**Figure 5:**
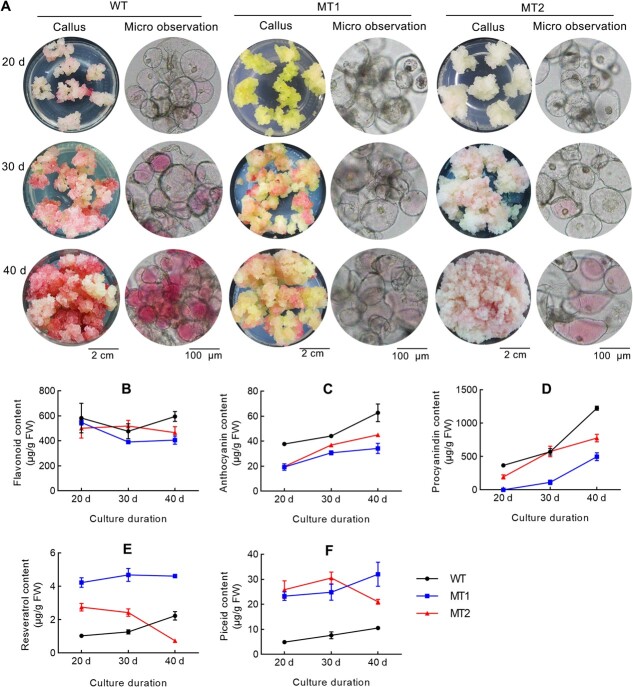
Phenotypes of mutant cell lines and determination of target metabolites at different culture durations. (A) Phenotypes and microscopic observations of *CHS2* mutant cell lines. Determination of (B) flavonoids, (C) anthocyanins, (D) proanthocyanindins, (E) resveratrol, and (F) piceid.

Based on prior metabolomic analysis, flavonoid biosynthesis was inhibited, and stilbenoid biosynthesis was promoted at 25 d. To quantitatively assess the level of target metabolites, flavonoids, anthocyanins, proanthocyanidins, resveratrol, and piceid were measured at 20, 30, and 40 d. The callus phenotype showed green and white-yellow calli in MT1 and MT2, respectively. As culture duration increased, a light-red tinge was observed on the callus surface. Microscopic observations indicated that anthocyanin accumulation was suppressed in mutant cell lines, consistent with the callus phenotype (Fig. 5A). WT and MT1 exhibited a similar pattern of flavonoid accumulation, decreasing at 30 d and then increasing at 40 d, whereas MT2 demonstrated an opposite pattern (Fig. 5B). Since many flavonoids are key substrates or precursors for downstream metabolite synthesis, continuous accumulation does not occur. Flavonoid content showed no significant difference between MTs and WT at 20 and 30 d. At 40 d, flavonoid content in WT, MT1 and MT2 was 594.5, 406.2, and 466.41 μg/g (fresh weight), significantly lower flavonoid content was observed in MTs compared to WT. The accumulation patterns of anthocyanin (Fig. 5C) and proanthocyanidin (Fig. 5D) differed from those of flavonoids, exhibiting a continuous increase due to their role as end-metabolites. In MT cell lines, the levels of anthocyanin and proanthocyanidin were significantly lower than in WT across various culture durations, peaking at 40 d. Specifically, anthocyanin content was 62.7, 34.25, 45.03 μg/g (fresh weight) in WT, MT1, and MT2, respectively, while proanthocyanidin content was 1225.33, 497.19, and 776.44 μg/g (fresh weight). This indicated that flavonoids, anthocyanins, and proanthocyanidins levels were notably reduced in MTs, particularly in MT1, due to *CHS2* mutations, which inhibited flavonoid accumulation. MT1 displayed a more pronounced suppressive effect than MT2, reflecting a greater impact of *CHS2* knockout.

Stilbenoids, including resveratrol and its derivative piceid, were also detected in both WT and MT cell lines (Fig. 5E and F). Notably, *CHS2* mutant callus cell lines showed a significant increase in stilbenoid levels compared to WT, except for resveratrol at 40 d. At 20 d, resveratrol content was 1.03, 4.22 and 2.76 μg/g (fresh weigh) in WT, MT1, and MT2, respectively, indicating a 4.1-fold and 2.7-fold increase in resveratrol levels in MT1 and MT2. Over a longer culture period, resveratrol content in WT increased to 1.3 and 2.2 μg/g at 30 and 40 d, respectively. In MT1, resveratrol levels stabilized at approximately 4.65 μg/g at 30 and 40 d in MT1, representing a 3.7 and 2.1-fold increase compared to WT. MT2 showed a 1.9-fold increase to 2.42 μg/g at 30 d, but levels decreased to 0.74 μg/g at 40 d. This dynamic equilibrium in resveratrol content suggests consumption during downstream biosynthesis of derivatives, such as piceid. Similarly, *CHS2* mutation resulted in significant piceid accumulation, with a 4.7-fold (23.27 μg/g) increase in MT1 and 5.3-fold (25.90 μg/g) increase in MT2 compared to WT (4.92 μg/g) at 20 d. Piceid levels further increased, showing a 3.26-fold (24.83 μg/g) and 4.0-fold (30.52 μg/g) rise in MT1 and MT2, respectively, compared to WT (7.62 μg/g). Piceid continued to accumulate, peaking at 32.06 μg/g in MT1, though declined to 21.01 μg/g in MT2, maintaining a 3.1-fold and 2.0-fold increase compared to WT (10.51 μg/g). In conclusion, *CHS2* mutation effectively enhanced resveratrol and piceid accumulation while inhibiting the accumulation of flavonoids, anthocyanins and proanthocyanidins.

### RNA-Seq revealed *CHS2* mutation significantly impacted the expression of target genes

**Figure 6:**
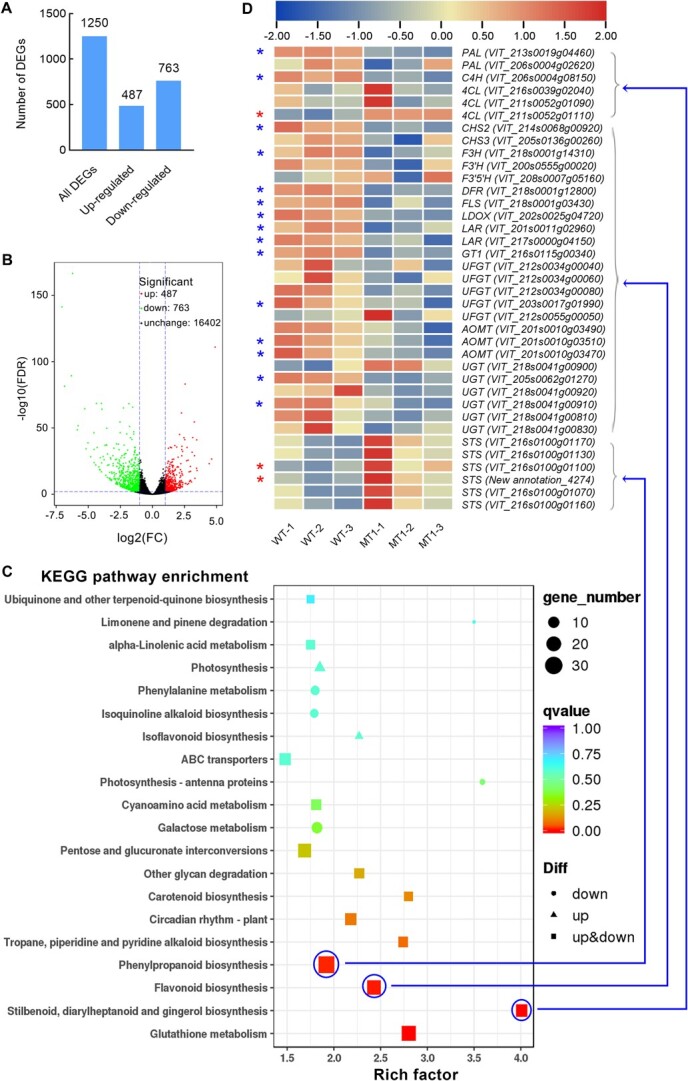
Analysis of differential expression genes (DEGs) and KEGG pathway enrichment in *CHS2* mutant cell line. (A) DEGs in MT1. A total of 1250 DEGs were identified in MT1. (B) Volcano map of DEGs in MT1. (C) KEGG pathway enrichment. (D) Cluster analysis of DEG involved in phenylpropanoid, flavonoid and stilbenoid biosynthesis. The *CHS2* mutation suppressed the expression of flavonoid biosynthesis-related genes while promoting STS expression. PAL: phenylalanine ammonialyase; C4H: Cinnamate 4-hydroxylase; 4CL: 4-coumarate:CoA ligase; CHI: Chalcone isomerase; F3H: flavanone hydroxylase; F3’5’H: flavanone 3′, 5′-hydroxylase; DFR: dihydroflavonol 4-reductase, FLS: flavonol synthase; LDOX: leucoanthocyanidin dioxygenase; LAR: leucoanthocyanidin reductase; GT1: anthocyanidin 5,3-O-glucosyltransferase; UFGT: UDP glucose - flavonoid 3–0-glucosyltransferase; AOMT: anthocyanidin O-methyltransferase; UGT: UDP-glucuronosyltransferase.

DAM and metabolite analyses demonstrated that the *CHS2* mutation suppressed flavonoid accumulation and enhanced stilbenoid biosynthesis, with MT1 showing more pronounced effects of the mutation. To further explore the gene expression profile involved in the target metabolic pathway, RNA-seq was conducted on WT and MT1 cell lines. CHS, a crucial enzyme in the biosynthesis of flavonoid and anthocyanin, utilizes the same substrates as STS in stilbenoid biosynthesis, potentially leading to a competitive interaction. RNA-seq yielded 6.19 Gb of clean data per sample with Q30 > 94.6% (Table S8), and 90.70% to 91.79% of the clean reads from the six samples were accurately mapped to the grape reference genome (Table S9), ensuring reliable RNA-seq results. The RNA-seq data have been deposited in the GAS database (Accession No. CRA017907). A total of 1250 differentially expressed genes (DEGs) were identified, comprising 487 upregulated and 763 downregulated DEGs in MT1 compared to WT (Fig. 6A and B). Gene ontology (GO) and clusters of orthologous groups (COG) results are shown in Figs S4 and S5. The MT1 cell line exhibited significantly enrichment in the phenylpropanoid, flavonoid, stilbenoid, and glutathione biosynthesis pathway (Fig. 6C). Glutathione S-transferase (GST), while not directly involved in flavonoid biosynthesis, plays a pivotal role in transport and modification of flavonoids. KEGG pathway enrichment analysis revealed that *CHS2* gene editing notably impacted target metabolic synthesis pathways.

The clustering heat map of DEGs showed distinct expression patterns of phenylpropanoids-related genes between WT and MT1 (Fig. 6D). *PAL* and *C4H* gene were downregulated, while *4CL* was upregulated. The other three phenylpropanoid genes exhibited no significant differences. *PAL*, *C4H*, and *4CL* are essential in phenylpropanoid biosynthesis, catalyzing the precursor formation for flavonoids and stilbenoids. In the flavonoid pathway, FPKM values indicated the suppression of many related genes. DEGs analysis revealed that 13 genes, including *CHS*, *F3H*, *DFR*, *FLS*, *LDOX*, *LAR*, *GT1*, *UFGT*, *AOMT*, and *UGT*, were downregulated, all of which are crucial in flavonoid, anthocyanin, and proanthocyanidin biosynthesis. No genes were upregulated, and 12 genes exhibited no significant differences. Consequently, the *CHS2* mutation resulted in the downregulation of key genes involved in flavonoid biosynthesis. In contrast, all six members of the *STS* gene family exhibited increased FPKM values, with two of them significantly upregulated during stilbenoid biosynthesis.

### *CHS2* mutation caused downregulation of genes associated with flavonoid synthesis but upregulation of *STS*

**Figure 7:**
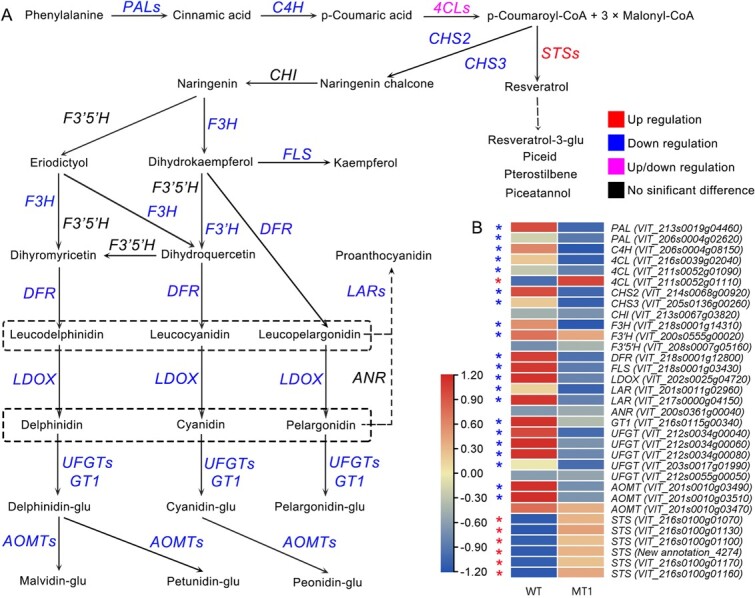
Gene expression in phenylpropanoid, flavonoid and stilbenoid biosynthesis. (A) Relative gene expression patterns in MT1 compared with WT in the target metabolic pathway. Most genes involved in flavonoid were downregulated, whereas six *STS* genes were up-regulated. (B) Cluster heat map analysis of relative gene expression. A total of 33 genes involved in phenylpropanoid, flavonoid, and stilbenoid biosynthesis were analyzed.

Transcriptome analysis confirmed that *CHS2* gene editing impacted gene expression within the targeted biosynthesis pathway. RT-qPCR assays conducted on WT and MT1 cell lines further validated the expression levels of genes involved in phenylpropanoid, flavonoid, and stilbenoid biosynthesis. The qPCR findings (Figs 7 and S6) aligned with prior transcriptome data (FPKM values), indicating consistent gene expression patterns. In phenylpropanoid biosynthesis, *CHS2* gene editing led to the downregulation of most genes in the MT1 mutant cell line, except for *4CL* (VIT_211S0062g01110). In flavonoid biosynthesis, 21 genes were verified by qPCR, revealing that 16 genes, including *CHS2*, *CHS3*, *F3H*, *F3’H*, *DFR*, *FLS*, *LDOX*, *LAR*, *GT1*, *UFGT*, *AOMT*, were downregulated, while the remaining five genes showed no significant difference between WT and MT1. For stilbenoid biosynthesis, all six tested *STS* genes exhibited significant upregulation in MT1, with a 5-to 10-fold increase in relative *STS* expression levels. These results indicate that the *CHS2* mutation promoted *STS* expression, which is essential for resveratrol biosynthesis, while most flavonoid biosynthesis-related genes were downregulated in MT1. Overall, *CHS2* gene editing caused the downregulation of crucial flavonoid biosynthesis genes but promoted the upregulation of *STS* genes.

### Integrated analysis revealed a metabolic pathway shift from flavonoids to stilbenoids due to *CHS2* mutation

**Figure 8:**
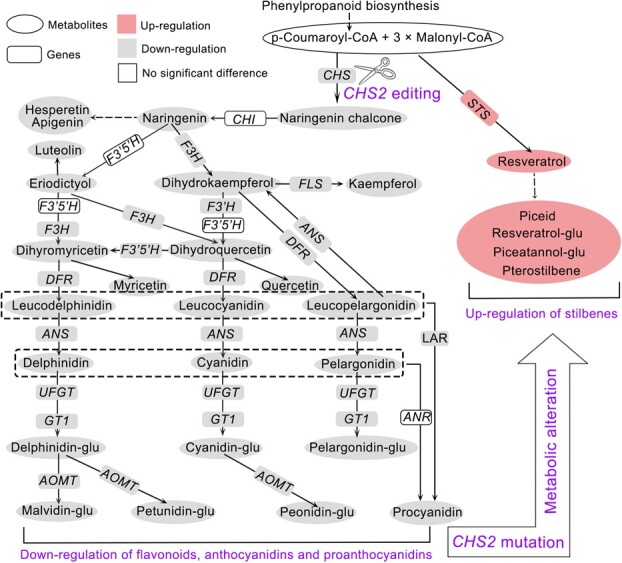
Integrated analysis of gene expression and metabolite accumulation. *CHS2* mutation suppressed gene expression and metabolite accumulation related to flavonoid, anthocyanin, and proanthocyanidin biosynthesis, while promoting gene expression and metabolite accumulation involved in stilbenoid biosynthesis, causing a metabolic pathway shift from flavonoids to stilbenoids.

In this study, *VdCHS2* genome editing was performed using CRISPR/Cas9 to generate *CHS2* mutant cell lines. An integrated analysis combining metabolomics, metabolite quantification, transcriptomics, and RT-qPCR was employed to elucidate the effects of *CHS2* genome editing on target metabolism (Fig. 8). The integrated analysis demonstrated that the C*HS2* mutation significantly affected the target metabolic pathways, including flavonoid and stilbenoid biosynthesis. DAM analysis revealed that flavonoid biosynthesis was suppressed, while stilbenoid biosynthesis was enhanced in *CHS2* mutant cell lines. The content of flavonoids, anthocyanins, and proanthocyanidins in *CHS2* mutant cell lines was significantly downregulated compared to WT cell line, whereas resveratrol and its derivatives were upregulated. The *CHS2* mutation facilitated an efficient accumulation of resveratrol and piceid but inhibited the accumulation of flavonoids, anthocyanins, and proanthocyanidins. Additionally, key genes involved in flavonoid metabolism were generally downregulated, whereas *STS* genes associated with stilbenoid (resveratrol) biosynthesis were upregulated. Consequently, the *CHS2* mutation suppressed the expression of genes related to flavonoid biosynthesis, but enhanced the expression of *STS* genes. In summary, the metabolite accumulation and gene expression patterns indicated pathway competition between flavonoids and stilbenoids. The *CHS2* mutation inhibited the expression of genes and accumulation of metabolites involved in flavonoid biosynthesis, while promoting those associated with stilbenoid production, thereby redirecting the flavonoid metabolic pathway towards stilbenoid biosynthesis.

## Discussion

### Targeted competitive gene suppression adds an efficient alternative strategy for promoting specific metabolic pathways

The genetic manipulation of structural and regulatory genes is widely used in plant metabolite synthesis. *STS* (*RS*) genes are responsible for the catalysis of one molecule of p-coumaroyl-CoA and three molecules of malonyl-CoA to resveratrol [35]. Overexpression of *STS* gene has proven effective in boosting resveratrol production. Previous research demonstrated that *VaSTS7* gene overexpression increased resveratrol content in transgenic *V. amurensis* grape cells by 3.2–6.6 times [36]. Similarly, *STS* transgenic suspension-cultured cells exhibited a two-fold increase in resveratrol accumulation compared to nontransgenic cell lines [37]. Transcription factors play key roles in resveratrol synthesis. For instance, VqWRKY53 recognizes and binds directly to the promoters of *VqSTS32* and *VqSTS41*, and its overexpression upregulated these genes, thereby enhancing the accumulation of trans-resveratrol and trans-piceid in *Vitis quinquangularis* [5]. Conversely, VvWRKY8 and VvMYB30 negatively regulated *VvSTS15/21* and resveratrol biosynthesis in grapevines, with *VvWRKY8* overexpression decreasing *VvSTS15/21* expression and resveratrol accumulation [38, 39]. However, RNAi-mediated downregulation of *MYB30* significantly increase resveratrol content in leaves and skin by reducing *STS* expression. Therefore, enhancing the expressions of target genes offers a common and typical approach to promoting specific metabolic pathways.

Suppressing competitive metabolic pathways can effectively redirect metabolic flux towards the synthesis of desired plant natural products, presenting a viable strategy for enhancing metabolite production. For example, the sterol pathway is a competing pathway for sesquiterpene artemisinin biosynthesis, previous research has shown that RNAi-silencing-based squalene synthase (SQS, a key enzyme of the sterol pathway) suppression in *Adonis autumnalis* led to a 3.14-fold increase in artemisinin content [40]. In *Nicotiana benthamiana*, RNAi silencing of both endogenous 5-epi-aristolochene synthase (*EAS*) and *SQS* resulted in a 2.8-fold increase in sesquiterpenoid (+)-valencene content by downregulating competing pathways [41]. Additionally, a seven-fold increase in isoflavone yield was achieved by suppressing flavanone 3-hydroxylase (*F3H*) involved in the flavanol pathway, which competes for naringenin as substrate with isoflavone synthase (*IFS*) [42].

In this study, we propose a novel approach based on CRISPR/Cas9 genome editing to suppress competitive metabolic pathways, resulting in enrichment of the target metabolites. Successful trials have demonstrated that knocking out two *CYP* genes (*CYP716A47* and *CYP716A53v2*) in *Panax ginseng* altered the saponin biosynthesis pathway [43]. Our findings revealed that stilbenoid biosynthesis competes with flavonoid biosynthesis, *CHS2* gene editing resulted in a decrease in flavonoids, anthocyanins, and proanthocyanidins, while promoting the accumulation of resveratrol and its derivatives. Thus, CRISPR/Cas9 based *CHS2* mutation is an effective strategy for promoting competitive target metabolite synthesis. Consequently, both target gene overexpression and CRISPR/Cas9-mediated competitive gene suppression are efficient strategies for targeted metabolite production.

### CRISPR/Cas9-mediated *CHS2* mutation shifts metabolism from flavonoids to stilbenoids

The CRISPR/Cas systems specifically recognize and cleave target DNA/RNA to produce double-strand breaks (DSB) [44], which trigger two independent endogenous DNA repair mechanisms: nonhomologous end joining (NHEJ) and homologous recombination (HR), resulting in chromosomal changes and genome editing [45]. The CRISPR/Cas9 system has thus far been effectively utilized in various woody species, including grape, litchi, pear, apple, and cacao [46, 47]. In this study, CRISPR/Cas9 induced numerous *CHS2* mutations, which were attributed to the complicated DNA repair mechanism after DSBs, consequently resulting in functional differentiation between the two *CHS2* mutations. CRISPR/Cas9 based *CHS2* editing generated multiple genetic mutations that contribute to phenotypic and physiological variation.

Two *CHS2* mutant cell lines demonstrated suppression of flavonoid accumulation, including anthocyanins, proanthocyanidins, quercetin, and kaempferol, and caused downregulation of genes involved in flavonoid biosynthesis including *CHS2*, *CHS3*, *F3H*, *F3’H*, *DFR*, *FLS*, and *LDOX*. Conversely, the expression of *STS* genes was upregulated during stilbenoid biosynthesis, which competes with the flavonoid pathway, significantly increasing stilbenoid content. A recent report highlighted the CHS/STS competition, the regulation competition in metabolic flow dominated by *CHS* and *STS* was responsible for the difference in accumulation trends of flavonoids and cajaninstilbene acid, indicating an inverse relationship between flavonoids and stilbenes [33]. In conclusion, CRISPR/Cas9-mediated *CHS2* mutations promoted the upregulation of target genes and metabolite accumulation, while concurrently suppressing competing genes and metabolite pathways, indicating a metabolic shift from flavonoids to stilbenoids.

### Comprehensive knockout and a 5′-region *CHS2* mutation explain the molecular mechanism for phenotype differentiation

The CRISPR/Cas9 system, is a powerful, highly efficient, convenient, and revolutionary tool for genome editing, can suffer from low efficiency due to suboptima sgRNA design [34]. Variations in sgRNA positions can lead to diverse gene-editing effects. Previously, a mutation in the first exon of *VvbZIP36*, a negative regulator in grapevines, has been documented to effectively suppress *VvbZIP36*, thereby indirectly enhancing the accumulation of target anthocyanins [48]. In our study, the MT1 cell line exhibited a more typical gene-knockout effect. Comprehensive *CHS2* knockout provided substantial evidence of significant phenotypic differentiation between MT1 (99.87% mutation) and MT2 (46.67% mutation) based on amplicon sequencing. Additionally, *CHS2* mutations led to premature termination of protein translation at ORF405–510 (5′-region) and ORF606–801 in MT1 and MT2, respectively. MT1 cells exhibited a more significant loss of *CHS2* function. Consequently, *CHS2* mutations downregulated key genes involved in flavonoid biosynthesis, particularly in MT1. The function differentiation between two *CHS2* mutations indicated that comprehensive gene knockout and 5′-region (upstream target position) mutations substantially resulted in loss of gene function.

Measurements of target metabolites demonstrated a significant increase in resveratrol and piceid in *CHS2* mutant cell lines. *CHS2* mutations caused downregulation of flavonoid biosynthesis and upregulation of stilbenoid biosynthesis, and *CHS2* mutant cell lines exhibited rapid gene upregulation and metabolite accumulation during early culture. In addition, MT1 and MT2 exhibited functional differentiation, which resulted from the extent of gene knockout and different target positions. The *CHS2 is* comprehensively knocked out in MT1, whereas the wild-type sequence remains the predominant genotype in the MT2 cell line. MT1 with a 5′-region mutation of *CHS2* exhibited significant resveratrol accumulation compared with MT2 and WT at 20 d, and maintained at a high level during extended periods of culture. Target selection was influenced by sequence specificity, PAM motif, GC content and secondary structure [49], and a 5′-region target sequence with PAM based on high sequence specificity, relative high GC content, and four or more consecutive T-free regions contributed high efficiency CRISPR/Cas genome editing [34]. Although recent reports have suggested that non-PAM sequences can be utilized in CRISPR/Cas-based genome editing [50, 51], the PAM structure remains predominantly employed in CRISPR/Cas9 applications. In conclusion, the amplicon sequencing offers a robust high-throughput approach for mutation screening, and is worth promoting for mutation detection following plant genome editing. Comprehensive knockout and a 5′-region *CHS2* mutation caused more severe loss of *CHS2* function and rapid stimulation of resveratrol accumulation.

### Genetic and cell engineering-based plant cell factory contributes high-yield production of resveratrol

Recently, food, medicine, and cosmetics industries have increasingly turned to natural products due to their lack of severe side effects [52]. Phytonutrients derived from natural plant products not only provide substantial physiological and pharmacological health benefits but also influence gene expression and epigenetic changes in humans [53, 54]. Resveratrol, a natural non-flavonoid polyphenol, is induced in response to biotic and abiotic stresses in a limited number of plant species [11]. The limited species availability, low resveratrol content, and complex extraction processes hinder the utilization of plant-derived resveratrol [16]. Our study aimed to obtain high-yield resveratrol callus cell lines via targeted regulation using CRISPR/Cas9 gene editing for *CHS2* knockout. Notably, resveratrol and its derivative piceid were significantly enhanced in the MT cell lines, achieving a 4.1-fold increase in resveratrol and a 5.3-fold increase in piceid. However, levels of stilbenoids, including resveratrol, are dynamic and fluctuate when exposed to abiotic and biotic stresses [55, 56]. Hence, an optimum culture protocol is essential to significantly enhance resveratrol biosynthesis using the high-yield cell line MT1. Various approaches can be employed, including suspension cell culture, metabolic precursor feeding, and the selection of bio- and abio-culture conditions. Consequently, high-yield cell lines, combined with an optimal culture protocol, present a viable solution for efficient natural resveratrol production. In conclusion, this study outlines the best practices for the targeted production of natural metabolites based on plant cell cultures and biosynthetic strategies. CRISPR/Cas9-based metabolic shifting facilitated resveratrol accumulation, providing new insights into metabolite biosynthesis and regulation. This research provides a foundation for addressing the challenges of high cost, long cycle-time, and low extraction efficiency in resveratrol production. Furthermore, competitive inhibition-based gene editing offers a novel breeding approach for eliminating non-target agronomic traits.

## Materials and methods

### Plant materials

During the fruit-setting period, *V. davidii* young grapes were harvested from Fuan Grape Germplasm Resource Nursery in Fujian, China. The immature embryos underwent surface sterilization by immersion in 75% alcohol for 30 s, followed by treatment with 8% sodium hypochlorite (NaClO) for 15 min, and were subsequently rinsed five times with sterile water. These immature embryos were then cultivated on Murashige and Skoog (MS) solid medium, which was supplemented with 1.0 mg/L 2,4-dichlorophenoxyacetic acid (2, 4-D), 3.0% sucrose and 0.6% agar (pH 5.8). Subculturing was performed every 20 d. The culture conditions were maintained at a temperature of 25 ± 2°C with a light intensity of 1800–2000 Lux under a photoperiod of 16 h light/8 h dark. A callus cell line, designed as “RXT29R” with a red phenotype, demonstrates the capability for anthocyanin biosynthesis.

### Target site selection and vector construction

*V. davidii* and *V. vinifera,* both classified under the *Vitis* genus, share orthologous gene sequences indicative of significant conservation within the genus. The *CHS2* target gene was cloned based on the *Vitis vinifera* reference genome (Assembly version: Genoscope.12X)*,* as reported in study [57], and the *CHS2* gene was deposited in GenBank (Accession No. OL906400). The pYLCRISPR/Cas9Pubi-H vector system [34] was used for *CHS2* gene editing. The CRISPR-GE online toolkit (http://skl.scau.edu.cn/home/) was used for target selection, primer design, and CRISPR/Cas9 vector construction. A high GC content(> 40%) was required to obtain a high-efficiency target sgRNA and to avoid using four or more consecutive T nucleotides in the target sequence. The potential off-target effects of the target sgRNAs were further evaluated using CRIPSR-P (http://cbi.hzau.edu.cn/cgi-bin/CRISPR). The primers used for vector construction are listed in Table S1. Overlapping PCR and golden gate cloning were performed, as previously described, to construct two single-target site CRISPR/Cas9 vectors (*VdCHS2*-lacZ-Atu3d) [34]. The recombinant plasmid was validated by PCR and sequencing and further introduced into *A. tumefaciens* GV3101 for subsequent plant transformation.

### Plant transformation and resistance selection

*Agrobacterium* cultured in liquid Luria-Bertani (LB) medium supplemented with 50 mg/L kanamycin (Kan) and 50 mg/L rifampicin (Rif) was harvested when OD_600_ reached 0.8–1.0, and resuspended in liquid MS medium to a final OD_600_ of 0.6 supplemented with 3.0% sucrose. Grape callus was incubated in *Agrobacterium* resuspensions for 10–12 min, subsequently cocultured on solid MS supplemented with 3.0% sucrose, 0.65% agar and 100 μmol/L acetosyringone (AS) for 60 h. The callus was then transferred to solid MS medium supplemented with 3.0% sucrose, 1.0 mg/L 2,4-D, 120 mg/L timentin (Tim), and 35 mg/L hygromycin (Hyg). Transgenic grape cells were selected based on Hyg resistance over three generations.

### Detection of *CHS2* mutation

Genomic DNA was extracted using the CTAB method [58] and used as a template for PCR amplification. Amplification was performed using primers flanking the target sites and putative off-target sites to generate PCR products (400–800 bp), which were sequenced directly using internal specific primers. The primers used for PCR amplification and mutation sequencing are listed in Table S1. PCR sequencing results were first decoded using DSDecodeM (http://skl.scau.edu.cn/dsdecode/) to obtain *CHS2* mutation types. Furthermore, PCR products were subcloned and 24 clones were sequenced to verify the mutation characteristics of the *CHS2* sequence.

### Amplicon sequencing analysis

Genomic DNA for PCR amplification was extracted as previously described. Primers ligated with adaptors were specifically designed to amplify DNA fragments of 250 bp and 259 bp for target1 and target2, respectively. The sequences of the primers are provided in Table S2. The PCR products were subjected to quality and concentration evaluation using agarose gel electrophoresis and Nanodrop. PCR libraries were constructed and amplicon sequencing was carried out by Tsingke (Beijing, China). Then the sequencing was executed on an Illumina Miseq platform (San Diego, USA) using pair-end 150 with a sequencing depth of 5000×. Amplicon sequencing data was submitted to the Genome Sequence Archive (GSA) of National Genomics Data Center (NGDC, https://ngdc.cncb.ac.cn/). Wild-type *CHS2* sequence served as the reference for mutation analysis.

### Non-targeted metabolomic analysis

Grape callus samples cultured for 25 d were freeze-dried using a vacuum freeze-dryer Scientz-100F (Scientz, Ningbo, Zhejiang, China) and crushed using a mixing mill (Retsch, Haan, Germany) for 90 s at 30 Hz. The dry powder (0.1 g) was dissolved in 1.2 mL 70% methanol and vortexed six times every 30 min, and subsequently placed at 4°C for 12 h. Extracts were then centrifuged at 12000 rcf at 4°C for 10 min, and the supernatant was then filtered using a 0.22 μm pore size membrane prior to UPLC-MS/MS detection. Chromatographic detection was performed using Biomarker Technologies (Beijing, China) and a Shimadzu Nexera X2 UHPLC system (Shimadzu Corporation, Kyoto, Japan) consisting of an Agilent SB-C18 column (2.1 mm × 100 mm, 1.8 μm). Metabolites eluted from the column were analyzed using an electrospray ionization (ESI)-triple quadrupole linear ion trap (QTRAP)-mass spectrometer (AB 4500 Q TRAP UPLC/MS/MS System). Chromatography and mass spectrometry were performed as previously described [59]. The MS data acquisition and processing were performed as previously described [60]. Relative quantification of metabolites was performed. The data were normalized prior to analysis using total peak area normalization. Metabolomics data were submitted to the OMIX database (https://ngdc.cncb.ac.cn/omix/) of the National Genomics Data Centerof National Genomics Data Center.

### Determination of flavonoid, anthocyanin, and proanthocyanidin

To further investigate the accumulation pattern of metabolites, WT and *CHS* mutant cell lines cultured for 20, 30, and 40 d were used for determination. Freeze-fresh callus samples from WT and MT cell lines were used for metabolite quantification. Flavonoids, anthocyanins, and proanthocyanidins were determined as previously described [61]. The flavonoid content assay was performed using the sodium nitrite-aluminum nitrate colorimetric method and normalized to rutin equivalents. Cyanidin 3-O-glucoside and proanthocyanidin were used as standards to normalize and evaluate the anthocyanin and proanthocyanidin content, respectively. HPLC purity (≥ 98%) of rutin, cyanidin 3-O-glucoside, and proanthocyanidin were provided by Solarbio (Beijing, China).

### Determination of resveratrol and piceid contents

Freeze-fresh callus samples of WT and MTs were ground in liquid nitrogen. Powder of 0.5 g from each sample was resuspended in 4 mL of methanol for resveratrol and piceid extraction, followed by ultrasonic disintegration for 20 min, followed by centrifugation at 4000 rcf for 20 min at 25°C. Residues were re-extracted from the supernatant using the steps outlined above. Total supernatant was stored in brown bottles and filtered using a 0.45 μm-pore-size membrane for further assay. A Thermo Scientific UltiMate 3000 UHPLC was used for quantitative analysis of resveratrol and piceid in grape cell lines using a Thermo Scientific Acclaim 120 C18 reverse phase column (4.6 × 250 mm, 5 μm, 120 A) to separate samples. The mobile phase comprised methanol (A) and water (B). The injection volume was 10 μL, the flow rate was 0.8 mL/min with the ratio of A:B as 40:60, and the temperature of the column oven was maintained at 30°C. The detection wavelength was 306 nm and the total running time was 50 min. Trans-resveratrol and piceid (Solarbio, Beijing, China, HPLC purity ≥98%) were used as standards. A gradient of trans-resveratrol solution (50, 25, 10, 5, 1, 0.2 ug/L) and piceid solution (40, 20, 10, 5, 1, 0.5 ug/L) were used to define the standard curve and calculate the linear regression equation.

### RNA-seq analysis

RNA was isolated using the RNAprep Pure Plant Plus Kit (Catalog No. DP441) with Polysaccharides & Polyphenolics-rich (Tiangen, Beijing, China), following the manufacturer’s protocol. The RNA quality was evaluated with a NanoDrop 2000 spectrophotometer (Thermo Fisher Scientific, Waltham, USA) and an Agilent 2100 Bioanalyzer (Agilent Technologies, Santa Clara, USA). Sequencing libraries were generated using the VAHTS Universal V6 RNA-seq Library Prep Kit for Illumina (Vazyme, Nanjing, China) according to the manufacturer’s recommendations. RNA sequencing was carried out on an Illumina NovaSeq 6000 platform (Illumina, San Diego, USA) by Biomarker Technologies (Beijing, China). Data quality was assessed using Q20, Q30, and GC content, adaptor and poly N sequences were removed, and low-quality reads were trimmed based on a Phred quality score threshold of ten. The raw RNA-Seq data was deposited in GSA. The reference genome for grapevine (Assembly version: Genoscope.12X, Annotation source: CRIBI, Annotation version: v2.1) was obtained from Phytozome (https://phytozome-next.jgi.doe.gov/info/Vvinifera_v2_1). Gene expression levels were quantified as fragments per kilobase of transcript per million fragments mapped (FPKM) using Cufflinks software [62]. Gene ontology (GO), clusters of orthologous groups (COG), and KEGG pathway enrichment analyses were performed as previously described [63–65]. Differential gene expression analysis was performed using DESeq [66], considering genes with absolute values of log2 ratio ≥ 2 and FDR significance scores <0.01.

### RT-qPCR analysis

Total RNA was isolated from transgenic cell lines and wild-type calli using the RNAprep Pure Plant Plus Kit, following the same protocol as mentioned above. The cDNA synthesis was conducted using a PrimeScript™ RT reagent Kit with gDNA Eraser (Takara, Otsu, Japan). Quantitative reverse transcription–PCR (RT-qPCR) was performed using TB Green® Premix Ex Taq™ (Takara, Otsu, Shiga, Japan) on LightCycler® 480 (Roche Applied Science, Basel, Switzerland). The PCR program was: pre-incubation at 95°C for 30 s, followed by 40 cycles of amplification at 95°C for 5 s and 60°C for 30 s. Then, the melting curve was conducted at 95°C for 5 s, 60°C for 60 s, and finally cooling at 50°C for 30 s. Each sample was run in triplicate. The primers used are listed in Table S2. Elongation factor1-α (*EF1-α*) was used as the reference gene according to the 2^−△△ct^ method to calculate the relative expression level.

### Statistical analyses

All experiments were performed in triplicate, and data are expressed as mean ± standard deviation (SD). Relative expression data and quantitative results of analyses of metabolites were analyzed using GraphPad Prism 8.0. One-way ANOVA was performed to determine significant differences (*p* < 0.05) between means. Genes with an absolute value of log2 ratio ≥ 2 and FDR significance score < 0.01 were used for differential expression gene (DEG) analyses. DAMs were screened with the following criteria: VIP > 1.0, fold changes ≥2 or fold changes ≤0.5.

## Supplementary Material

Web_Material_uhae268

## Data Availability

Data supporting the results are available in the article and its supplementary data.
