# Supplementary material for: CRISPR/Cas9-mediated CHS2 mutation provides a new insight into resveratrol biosynthesis by causing a metabolic pathway shift from flavonoids to stilbenoids in Vitis davidii cells
Source: Hortic Res. 2024 Oct 9;12(1):uhae268. doi: 10.1093/hr/uhae268 (PMC11718387; doi:10.1093/hr/uhae268)
Supplement: Web_Material_uhae268 [file web_material_uhae268.zip › Supplementary material.docx]

Supplementary material

**CRISPR/Cas9 mediated *CHS2* mutation provides a new insight into resveratrol biosynthesis by causing a metabolic pathway shift from flavonoids to stilbenoids in *Vitis davidii* cells**

**Table S1.** Primers for CRISPR/Cas9-*CHS2* vector construction and mutation detection

| **Primer name** | **Primer sequence (5’-3’)** | **Vector or purpose** |
| --- | --- | --- |
| gRT1-F | GGGCATGTCAACACCGGAGGGTTTTAGAGCTAGAAAT | CRISPR/Cas9 vector construction for two *CHS2* single-target |
| AtU3dT1-R | CCTCCGGTGTTGACATGCCCTGACCAATGGTGCTTTG |  |
| gRT2-F | GAATCCAGGTGGGTGTCAGAGTTTTAGAGCTAGAAAT |  |
| AtU3dT2-R | TCTGACACCCACCTGGATTCTGACCAATGGTGCTTTG |  |
| U-F | CTCCGTTTTACCTGTGGAATCG |  |
| gR-R | CGGAGGAAAATTCCATCCAC |  |
| Pps-GGL | TTCAGAGGTCTCGACTAGTATGGAATCGGCAGCAAAGG |  |
| Pgs-GGR | AGCGTGGGTCTCGACCGACGCGTATCCATCCACTCCAAGCTC |  |
| MT1-F1 | GGAGAACCCCAACGTCTGTGCCTA | PCR mutation detection for *CHS2* target1 |
| MT1-R1 | AATGGAACGTGAGACCCACTTCGC |  |
| MT1-F2 | GTCTGTGCCTACATGGCCCCATCT | Sequencing identification for *CHS2* target1 |
| MT1-R2 | GAGTGGGCGCTCGATTTTGGTATC |  |
| MT2-F1 | ATTATCATTGGTGCAGACCCAGAT | PCR mutation detection for *CHS2* target2 |
| MT2-R1 | ATTTCGTCCAGGATAAACAGCACG |  |
| MT2-F2 | CTCAGAACGTGTCGAGTTGCTCTC | Sequencing identification for *CHS2* target2 |
| MT2-R2 | TGCAGACCCAGATACCAAAATCGAG |  |
| CHS3-M-F1 | TTGACAGAGGAGATCCTGAAAG | PCR for target1 off-target detection in *CHS3* |
| CHS3-M-R1 | TGTGGAGCGTAACTTCTCGGG |  |
| CHS3-M-F2 | AGCTTCCCTGGATGCTAGGCAG | Sequencing for target1 off-target detection in *CHS3* |
| CHS3-M-R2 | GGGCTTTAGGGCTAATTTTTC |  |
| NGS-CHS2-T1-F | TCGTCGGCAGCGTCAGATGTGTATAAGAGACAG  TGGCAAGGAAGCTGCTGTCAAGG | PCR of *CHS2* target1 for Amplicon sequencing  (primer with adaptor) |
| NGS-CHS2-T1-R | GTCTCGTGGGCTCGGAGATGTGTATAAGAGACAG  GACTAAAACACGGGCGCCGGCGT |  |
| NGS-CHS2-T2-F | TCGTCGGCAGCGTCAGATGTGTATAAGAGACAG  CAAACCCTCCGTCAAGAGGCTGA | PCR of *CHS2* target2 for Amplicon sequencing  (primer with adaptor) |
| NGS-CHS2-T2-R | GTCTCGTGGGCTCGGAGATGTGTATAAGAGACAG  GAGTGGGCGCTCGATTTTGGTATC |  |

**Table S2.** Primers for real-time qPCR

| **Primer name** | **Primer sequence (5’-3’)** | **Primer name** | **Primer sequence (5’-3’)** |
| --- | --- | --- | --- |
| PAL-2620-F | TCACACCACAACGGCAAC | F3'5'H-5160-F | TTTCTCCAACCGTCCACC |
| PAL-2620-R | CCACCATTCTCTTCACCTCC | F3'5'H-5160-R | GCCTGCTCAACTCACACA |
| PAL-4460-F | GGCGAGAGAGGGAGTAAAGG | DFR-2800-F | TGCTGATGAAGGAAGTTTCG |
| PAL-4460-R | GGCAGTGTGTGGCAAGATT | DFR-2800-R | GCGGAGGATGTGAATACAAG |
| C4H-8150-F | ACTTGAACCACCTGAACCTC | FLS-3430-F | CGGTTTGGAAGGGAAGGTC |
| C4H-8150-R | ATCCGAACTCCACTCCCT | FLS-3430-R | GCCTGGAACATCATTGGG |
| 4CL-1090-F | CTGCGGATTGAGAGTTGG | LDOX-4720-F | GGAAGTTGGTGGGATGGA |
| 4CL-1090-R | TGATGGAGGAAAGGTCGT | LDOX-4720-R | GTGGAGGATGAAGGTGAGAG |
| 4CL-1110-F | GACATCAACATCCCGAGTC | LAR-2960-F | GGTTTCATTGGTCAGTTCGT |
| 4CL-1110-R | GCCGAGAAATGAGAAGACA | LAR-2960-R | GCCTCCTGCTCGTTTATCA |
| 4CL-2040-F | GCAGCCCTTTCCTACTAAGTC | LAR-4150-F | CAATACCCACCCTGCTGA |
| 4CL-2040-R | TCATCATCGTCAACATAGCC | LAR-4150-R | TTCTCCCACAGTGATGCC |
| STS-1070-F | TGATGGGTCTTCTGCTGTG | ANR-0040-qF | GCAGAAGGGCTATGCTGTCA |
| STS-1070-R | CAATGGCTCCTGCTGAAT | ANR-0040-qR | AGACAAAGTCGCAACCTGCT |
| STS-1110-F | AAGGAAGCAGCACTGAAGG | GT1-0340-F | GTTGGCGGTTTCGTGACT |
| STS-1110-R | AGCACCAGGCATTTCTACAC | GT1-0340-R | ATCCTTTGCTCGGCTTGC |
| STS-1160-F | CCGACCACTGTGTCTACCA | UFGT-1990-F | ATCGCATCTCCGTCACTG |
| STS-1160-R | CAATGTTTGGGTGCTCCTC | UFGT-1990-R | GTGGACAAACCGAATCCG |
| STS-4274-F | GGTAAGGAAGCAGCACTGAAG | UFGT-0060-F | CTGCTGTTCTTGGCTTCC |
| STS-4274-R | AGCACCAGGCATTTCTACAC | UFGT-0060-R | CCTCCGCTTTCCTTGTCA |
| STS-1170-F | CCATCAAGGGTGCTATGC | UFGT-0050-F | TCTCTTCTTCACTTCCAGCG |
| STS-1170-R | CAAGTTGGAAGAGTGGTCGT | UFGT-0050-R | CATAACTCGGACCTCCAGC |
| STS-1130-F | CCGACCACTGTGTCTACCA | UFGT-0040-F | TGCTGAGTTGGGTGTTCTG |
| STS-1130-R | CAATGTTTGGGTGCTCCTC | UFGT-0040-R | GGGTGTTGAGTATGGGTCC |
| CHS2-0920-F | GTCCCAGGGTTGATTTCC | UFGT-0080-F | GCTGTTCTTGGCTTCCTGTT |
| CHS2-0920-R | GCTCTCAGTTTCTCTTCCTTCA | UFGT-0080-R | CCCTCCGCTTACCTTGTCA |
| CHS3-0260-F | CCCGTGTCCTTGTTGTCTG | AOMT-3510-F | CGGTTATTCTCTCTTGGCAAC |
| CHS3-0260-R | GATTGTCTGGGCTGCTGA | AOMT-3510-R | CCTTCTTCTTTCCCATCGG |
| CHI-3820-qF | GTTCCTACGCTCGCCGTCAA | AOMT-3470-F | GAATGTGCCTGTTGACGA |
| CHI-3820-qR | ATACTGGCGACCCGTCAAAG | AOMT-3470-R | TTGCCTTGCCATCTTGAG |
| F3H-4310-F | GAAGATTGTGGAGGCGTGT | AOMT-3490-F | GGAACGAAGAAGGGACATT |
| F3H-4310-R | TGGAGATGACTGGAGACGA | AOMT-3490-R | CGCTACTGAACCAAACCAT |
| F3'H-0020-F | GGAAAGCAGCAACAGCAG | EF1-α-qF | CGCCTGTCAATCTTGGTCAGTAT |
| F3'H-0020-R | GCGAGGTCAGCAAAGTAGG | EF1-α-qR | AATGGCTATGCCCCTGTTCTG |

**Table S3.** Data statistics of amplicon sequencing

| Sample | ReadSum | BaseSum | GC(%) | Q20(%) | Q30(%) |
| --- | --- | --- | --- | --- | --- |
| MT1-WT | 6133 | 1852166 | 57.89 | 98.03 | 97.34 |
| MT1-Sample1 | 4599 | 1388898 | 57.62 | 97.83 | 97.13 |
| MT1-Sample2 | 5833 | 1761566 | 57.67 | 97.7 | 96.94 |
| MT1-Sample3 | 5406 | 1632612 | 57.61 | 97.89 | 97.22 |
| MT2-WT | 5205 | 1571910 | 57.96 | 95.95 | 94.23 |
| MT2-Sample1 | 6004 | 1813208 | 58.16 | 96.3 | 94.75 |
| MT2-Sample2 | 5827 | 1759754 | 58.11 | 96.02 | 94.41 |
| MT2-Sample3 | 5220 | 1576440 | 58.17 | 95.76 | 94.07 |

**Table S4.** Mutation information of *CHS2* target1

| Sample | Mutation | Type | Position | Sequence | Count | Ratio(%) |
| --- | --- | --- | --- | --- | --- | --- |
| Sample 1 | WT | - | - | - | 8 | 0.18 |
|  | 1 | I(+1) | 397 | A | 2760 | 61.28 |
|  | 2 | D(-4) | 397-400 | TCCG | 1447 | 32.13 |
|  | 3 | D(-8) | 397-404 | TCCGGTGT | 167 | 3.71 |
|  | 4 | D(-4) | 398-401 | CCGG | 69 | 1.53 |
|  | 5 | D(-8) | 398-405 | CCGGTGTT | 48 | 1.07 |
|  | 6 | I(+1); D(-15) | 397;  D:409-423 | A;  ATGCCCGGTGCTGAC | 4 | 0.09 |
|  | 7 | D(-14) | 398-411 | CCGGTGTTGACATG | 1 | 0.02 |
| Sample 2 | WT | - | - | - | 2 | 0.03 |
|  | 1 | I(+1) | 397 | A | 3687 | 63.77 |
|  | 2 | D(-4) | 397-400 | TCCG | 1917 | 33.15 |
|  | 3 | D(-8) | 397-404 | TCCGGTGT | 75 | 1.30 |
|  | 4 | D(-4) | 398-401 | CCGG | 48 | 0.83 |
|  | 5 | D(-8) | 398-405 | CCGGTGTT | 48 | 0.83 |
|  | 6 | I(+1); D(-15) | 397; 409-423 | A; ATGCCCGGTGCTGAC | 3 | 0.05 |
|  | 7 | I(+1); D(-15) | 397; 406-420 | A; GACATGCCCGGTGCT | 1 | 0.02 |
|  | 8 | D(-14) | 398-411 | CCGGTGTTGACATG | 1 | 0.02 |
| Sample 3 | WT | - | - | - | 9 | 0.17 |
|  | 1 | I(+1) | 397 | A | 3416 | 64.48 |
|  | 2 | D(-4) | 397-400 | TCCG | 1554 | 29.33 |
|  | 3 | D(-8) | 397-404 | TCCGGTGT | 168 | 3.17 |
|  | 4 | D(-4) | 398-401 | CCGG | 81 | 1.53 |
|  | 5 | D(-8) | 398-405 | CCGGTGTT | 66 | 1.25 |
|  | 6 | D(-4) | 395-398 | CCTC | 1 | 0.02 |
|  | 7 | I(+1) | 398 | G | 1 | 0.02 |
|  | 8 | D(-4) | 399-402 | CGGT | 1 | 0.02 |
|  | 9 | I(+1) | 399 | C | 1 | 0.02 |

I: Insertion;

D: Deletion.

**Table S5. Mutation information of *CHS2* target2**

| Sample | Mutation | Type | Position | Sequence | Count | Ratio(%) |
| --- | --- | --- | --- | --- | --- | --- |
| Sample 1 | WT | - | - | - | 3202 | 53.55 |
|  | 1 | I(+1) | 606 | G | 1716 | 28.70 |
|  | 2 | I(+1) | 606 | T | 508 | 8.50 |
|  | 3 | D(-1) | 607 | G | 483 | 8.08 |
|  | 4 | S(1) | 610 | A->G; | 44 | 0.74 |
|  | 5 | S(1) | 608 | A->T; | 8 | 0.13 |
|  | 6 | S(1) | 605 | C->T; | 5 | 0.08 |
|  | 7 | D(-3) | 607-609 | GAC | 4 | 0.07 |
|  | 8 | I(+1) | 606 | A | 4 | 0.07 |
|  | 9 | S(1) | 607 | G->A; | 2 | 0.03 |
|  | 10 | I(+1);D(-1) | 605;614 | T; A | 1 | 0.02 |
|  | 11 | D(-1) | 606 | T | 1 | 0.02 |
|  | 12 | D(-1) | 608 | A | 1 | 0.02 |
| Sample 2 | WT | - | - | - | 3031 | 52.73 |
|  | 1 | I(+1) | 606 | G | 1575 | 27.40 |
|  | 2 | D(-1) | 607 | G | 545 | 9.48 |
|  | 3 | I(+1) | 606 | T | 525 | 9.13 |
|  | 4 | D(-5) | 607-611 | GACAC | 33 | 0.57 |
|  | 5 | S(1) | 608 | A->G; | 11 | 0.19 |
|  | 6 | S(1) | 604 | T->C; | 5 | 0.09 |
|  | 7 | S(1) | 605 | C->T; | 4 | 0.07 |
|  | 8 | S(1) | 606 | T->G; | 4 | 0.07 |
|  | 9 | S(1) | 607 | G->T; | 4 | 0.07 |
|  | 10 | D(-1) | 608 | A | 3 | 0.05 |
|  | 11 | I(+1) | 608 | A | 2 | 0.03 |
|  | 12 | I(+1) | 605 | G | 1 | 0.02 |
|  | 13 | D(-4) | 607-610 | GACA | 1 | 0.02 |
|  | 14 | I(+1) | 606 | A | 4 | 0.07 |
| Sample 3 | WT | - | - | - | 2799 | 53.72 |
|  | 1 | I(+1) | 606 | G | 1513 | 29.04 |
|  | 2 | D(-1) | 607 | G | 445 | 8.54 |
|  | 3 | I(+1) | 606 | T | 440 | 8.45 |
|  | 4 | D(-1) | 608 | A | 4 | 0.08 |
|  | 5 | I(+1) | 607 | A | 3 | 0.06 |
|  | 6 | D(-4) | 607-610 | GACA | 2 | 0.04 |
|  | 7 | D(-11) | 601-611 | CCCTCTGACAC | 1 | 0.02 |
|  | 8 | I(+1) | 604 | G | 1 | 0.02 |
|  | 9 | D(-5) | 608-612 | ACACC | 1 | 0.02 |
|  | 10 | I(+1) | 607 | T | 1 | 0.02 |

I: Insertion;

D: Deletion;

S: Substitution.

**Table S6.** Differential accumulation metabolites (DAMs) involved in stilbenoid and flavonoid pathways in MT1

| Metabolites | WT-1 | WT-2 | WT-3 | MT1-1 | MT1-2 | MT1-3 | Fold Change(FC) | log2FC | VIP | Regulated |
| --- | --- | --- | --- | --- | --- | --- | --- | --- | --- | --- |
| Resveratrol-3,5-di-O-glucoside | 0.000026 | 0.000015 | 0.000018 | 0.000089 | 0.000060 | 0.000062 | 3.56 | 1.85 | 1.23 | up |
| Piceatannol-3'-O-glucoside | 0.003933 | 0.001910 | 0.002983 | 0.007249 | 0.005572 | 0.007275 | 2.28 | 1.24 | 1.21 | up |
| Piceid | 0.000327 | 0.000229 | 0.000285 | 0.000648 | 0.000620 | 0.000517 | 2.12 | 1.09 | 1.20 | up |
| Pterostilbene | 0.000000 | 0.000000 | 0.000000 | 0.000007 | 0.000005 | 0.000002 | 11.46 | 3.38 | 1.10 | up |
| Cyanidin-3-O-(6''-O-acetyl)glucoside | 0.000596 | 0.000313 | 0.000459 | 0.000250 | 0.000173 | 0.000234 | 0.48 | -1.03 | 1.07 | down |
| Cyanidin-3-O-(6''-O-caffeoyl)glucoside | 0.000261 | 0.000300 | 0.000149 | 0.000151 | 0.000101 | 0.000096 | 0.49 | -1.00 | 1.03 | down |
| Cyanidin-3-O-(6''-O-p-Coumaroyl)glucoside | 0.034795 | 0.034733 | 0.030649 | 0.017656 | 0.012078 | 0.012620 | 0.42 | -1.26 | 1.29 | down |
| Malvidin-3,5-di-O-glucoside (Malvin) | 0.001013 | 0.000837 | 0.001667 | 0.000290 | 0.000042 | 0.000014 | 0.10 | -4.33 | 1.18 | down |
| Malvidin-3-O-(6''-O-p-coumaroyl)glucoside | 0.000786 | 0.000785 | 0.001005 | 0.000038 | 0.000014 | 0.000002 | 0.02 | -6.47 | 1.30 | down |
| Malvidin-3-O-(6''-O-p-coumaroyl)glucoside-5-O-glucoside | 0.000498 | 0.000530 | 0.000724 | 0.000107 | 0.000021 | 0.000003 | 0.08 | -4.88 | 1.27 | down |
| Peonidin-3-O-(6''-O-Acetyl)glucoside | 0.000603 | 0.000417 | 0.000495 | 0.000115 | 0.000070 | 0.000035 | 0.15 | -2.92 | 1.27 | down |
| Peonidin-3-O-(6''-O-caffeoyl)glucoside | 0.000248 | 0.000266 | 0.000152 | 0.000096 | 0.000053 | 0.000021 | 0.26 | -2.18 | 1.18 | down |
| Peonidin-3-O-(6''-O-p-coumaroyl)glucoside | 0.036469 | 0.037956 | 0.035625 | 0.009008 | 0.003493 | 0.001311 | 0.13 | -3.41 | 1.31 | down |
| Peonidin-3-O-(6''-O-p-Coumaroylglucoside)-5-O-Glucoside | 0.004426 | 0.007164 | 0.008612 | 0.003369 | 0.001488 | 0.000503 | 0.27 | -2.25 | 1.13 | down |
| Peonidin-3-O-glucoside | 0.026342 | 0.024426 | 0.026136 | 0.006254 | 0.002643 | 0.002025 | 0.14 | -2.99 | 1.31 | down |
| Peonidin-3-O-rutinoside | 0.000020 | 0.000026 | 0.000013 | 0.000004 | 0.000007 | 0.000014 | 0.42 | -1.38 | 1.01 | down |
| Peonidin-3-O-sambubioside | 0.000043 | 0.000047 | 0.000064 | 0.000030 | 0.000008 | 0.000005 | 0.28 | -2.20 | 1.16 | down |
| Delphinidin-3-O-(6''-O-p-coumaroyl)glucoside | 0.000171 | 0.000217 | 0.000155 | 0.000038 | 0.000022 | 0.000011 | 0.13 | -3.10 | 1.28 | down |
| Pelargonidin-3-O-glucoside | 0.000459 | 0.000476 | 0.000426 | 0.000136 | 0.000105 | 0.000169 | 0.30 | -1.75 | 1.31 | down |
| Proanthocyanidins | 0.000068 | 0.000065 | 0.000061 | 0.000033 | 0.000023 | 0.000017 | 0.38 | -1.45 | 1.28 | down |
| Procyanidin A2 | 0.000025 | 0.000028 | 0.000040 | 0.000003 | 0.000006 | 0.000002 | 0.12 | -3.10 | 1.25 | down |
| Procyanidin B1 | 0.000274 | 0.000278 | 0.000193 | 0.000034 | 0.000057 | 0.000095 | 0.25 | -2.11 | 1.24 | down |
| Procyanidin B2 | 0.003035 | 0.002593 | 0.002043 | 0.000313 | 0.000516 | 0.000519 | 0.18 | -2.53 | 1.27 | down |
| Procyanidin B3 | 0.000395 | 0.000360 | 0.000267 | 0.000052 | 0.000080 | 0.000102 | 0.23 | -2.16 | 1.26 | down |
| Procyanidin B4 | 0.000665 | 0.000659 | 0.000461 | 0.000066 | 0.000098 | 0.000124 | 0.16 | -2.66 | 1.27 | down |
| Procyanidin C1 | 0.000434 | 0.000392 | 0.000240 | 0.000022 | 0.000044 | 0.000048 | 0.11 | -3.26 | 1.24 | down |
| Procyanidin C1 3'-O-gallate | 0.000045 | 0.000028 | 0.000016 | 0.000000 | 0.000005 | 0.000009 | 0.16 | -5.47 | 1.08 | down |
| Procyanidin C2 | 0.000227 | 0.000170 | 0.000121 | 0.000008 | 0.000023 | 0.000019 | 0.10 | -3.47 | 1.23 | down |
| Catechin gallate | 0.001289 | 0.001603 | 0.000738 | 0.000154 | 0.000330 | 0.000330 | 0.22 | -2.17 | 1.15 | down |
| Catechin-catechin-catechin | 0.000513 | 0.000351 | 0.000245 | 0.000019 | 0.000042 | 0.000041 | 0.09 | -3.48 | 1.20 | down |
| Epicatechin | 0.021041 | 0.020437 | 0.017291 | 0.003406 | 0.004249 | 0.004353 | 0.20 | -2.29 | 1.30 | down |
| Epicatechin glucoside | 0.000356 | 0.000460 | 0.000380 | 0.000046 | 0.000046 | 0.000040 | 0.11 | -3.17 | 1.30 | down |
| Epicatechin-epiafzelechin | 0.000022 | 0.000022 | 0.000018 | 0.000001 | 0.000003 | 0.000002 | 0.09 | -3.64 | 1.30 | down |
| (-)-Epicatechin gallate | 0.001363 | 0.001648 | 0.000814 | 0.000141 | 0.000300 | 0.000370 | 0.21 | -2.29 | 1.18 | down |
| Apigenin-3'-O-α-D-glucopyranoside | 0.000066 | 0.000050 | 0.000047 | 0.000015 | 0.000014 | 0.000020 | 0.30 | -1.73 | 1.25 | down |
| Apigenin-6-C-(2''-glucuronyl)xyloside | 0.000100 | 0.000088 | 0.000056 | 0.000005 | 0.000013 | 0.000014 | 0.13 | -3.03 | 1.23 | down |
| Apigenin-7-O-(6''-p-Coumaryl)glucoside | 0.000596 | 0.000889 | 0.000638 | 0.000102 | 0.000092 | 0.000066 | 0.12 | -3.03 | 1.27 | down |
| Apigenin-7-O-neohesperidoside (Rhoifolin) | 0.000248 | 0.000363 | 0.000325 | 0.000067 | 0.000057 | 0.000017 | 0.15 | -2.94 | 1.27 | down |
| Apigenin-7-O-rutinoside (Isorhoifolin) | 0.000545 | 0.000813 | 0.000611 | 0.000138 | 0.000110 | 0.000050 | 0.15 | -2.82 | 1.26 | down |
| Dihydrocharcone-4'-O-glucoside | 0.000066 | 0.000079 | 0.000049 | 0.000027 | 0.000023 | 0.000022 | 0.38 | -1.39 | 1.21 | down |
| Dihydrokaempferide | 0.000043 | 0.000029 | 0.000038 | 0.000021 | 0.000006 | 0.000012 | 0.36 | -1.65 | 1.17 | down |
| Dihydrokaempferol-3-O-glucoside | 0.000761 | 0.000724 | 0.000803 | 0.000319 | 0.000249 | 0.000572 | 0.50 | -1.09 | 1.17 | down |
| Dihydrokaempferol-7-O-glucoside | 0.000234 | 0.000198 | 0.000192 | 0.000086 | 0.000065 | 0.000109 | 0.42 | -1.29 | 1.26 | down |
| Eriodictyol 7-O-β-D-glucopyranoside | 0.000799 | 0.000758 | 0.000797 | 0.000345 | 0.000273 | 0.000515 | 0.48 | -1.10 | 1.24 | down |
| Eriodictyol-8-C-glucoside | 0.001235 | 0.001134 | 0.001096 | 0.000314 | 0.000326 | 0.000415 | 0.30 | -1.73 | 1.31 | down |
| Galangin-7-O-glucoside | 0.000048 | 0.000048 | 0.000026 | 0.000000 | 0.000000 | 0.000000 | 0.00 | -12.72 | 1.24 | down |
| Hesperetin-5-O-glucoside | 0.004781 | 0.005106 | 0.005095 | 0.001925 | 0.001548 | 0.001628 | 0.34 | -1.56 | 1.31 | down |
| Hesperetin-6-C-glucoside-7-O-glucoside | 0.000460 | 0.000491 | 0.000514 | 0.000316 | 0.000180 | 0.000205 | 0.48 | -1.11 | 1.25 | down |
| Hesperetin-7-O-glucoside | 0.000398 | 0.000343 | 0.000367 | 0.000081 | 0.000042 | 0.000092 | 0.19 | -2.45 | 1.31 | down |
| Hesperetin-8-C-glucoside-3'-O-glucoside | 0.000196 | 0.000178 | 0.000189 | 0.000092 | 0.000081 | 0.000053 | 0.40 | -1.36 | 1.29 | down |
| Hispidulin-7-O-Glucoside | 0.000101 | 0.000182 | 0.000171 | 0.000088 | 0.000041 | 0.000024 | 0.34 | -1.74 | 1.11 | down |
| Kaempferol-3,7-di-O-glucoside | 0.000282 | 0.000319 | 0.000200 | 0.000188 | 0.000102 | 0.000097 | 0.48 | -1.09 | 1.10 | down |
| Kaempferol-3,7-O-dirhamnoside (Kaempferitrin) | 0.000504 | 0.000430 | 0.000319 | 0.000046 | 0.000073 | 0.000099 | 0.17 | -2.57 | 1.26 | down |
| Kaempferol-3-O-(6''-p-Coumaroyl)glucoside (Tiliroside) | 0.000040 | 0.000052 | 0.000029 | 0.000016 | 0.000008 | 0.000010 | 0.27 | -1.89 | 1.19 | down |
| Kaempferol-3-O-galactoside (Trifolin) | 0.000104 | 0.000086 | 0.000083 | 0.000018 | 0.000020 | 0.000032 | 0.25 | -2.02 | 1.29 | down |
| Kaempferol-3-O-glucorhamnoside | 0.033654 | 0.035255 | 0.031021 | 0.019409 | 0.010154 | 0.013442 | 0.43 | -1.27 | 1.26 | down |
| Kaempferol-3-O-neohesperidoside | 0.038414 | 0.041159 | 0.034743 | 0.019006 | 0.012872 | 0.013956 | 0.40 | -1.34 | 1.29 | down |
| Kaempferol-3-O-rutinoside(Nicotiflorin) | 0.034121 | 0.036454 | 0.029662 | 0.017837 | 0.011299 | 0.013718 | 0.43 | -1.25 | 1.27 | down |
| Luteolin-4'-O-glucoside | 0.000095 | 0.000166 | 0.000177 | 0.000038 | 0.000013 | 0.000019 | 0.16 | -2.73 | 1.21 | down |
| Luteolin-7,3'-di-O-glucoside | 0.000297 | 0.000331 | 0.000201 | 0.000168 | 0.000127 | 0.000090 | 0.46 | -1.12 | 1.13 | down |
| Luteolin-7-O-(6''-caffeoyl)rhamnoside | 0.000041 | 0.000050 | 0.000031 | 0.000014 | 0.000009 | 0.000012 | 0.28 | -1.82 | 1.23 | down |
| Naringenin-4'-O-glucoside | 0.000279 | 0.000237 | 0.000216 | 0.000146 | 0.000106 | 0.000103 | 0.49 | -1.05 | 1.24 | down |
| Naringenin-7-O-glucoside (Prunin) | 0.001434 | 0.001529 | 0.001355 | 0.000552 | 0.000612 | 0.000690 | 0.43 | -1.22 | 1.30 | down |
| Quercetin | 0.000033 | 0.000022 | 0.000030 | 0.000014 | 0.000012 | 0.000015 | 0.48 | -1.05 | 1.19 | down |
| Quercetin-3-O-(2''-O-glucosyl)glucuronide | 0.000284 | 0.000519 | 0.000403 | 0.000092 | 0.000077 | 0.000083 | 0.21 | -2.21 | 1.21 | down |
| Quercetin-3-O-(6''-p-Coumaroyl)galactoside | 0.001405 | 0.001628 | 0.000951 | 0.000243 | 0.000103 | 0.000064 | 0.10 | -3.47 | 1.25 | down |
| Quercetin-3-O-galactoside (Hyperin) | 0.008243 | 0.006805 | 0.007255 | 0.003495 | 0.002827 | 0.002586 | 0.40 | -1.33 | 1.29 | down |
| Quercetin-3-O-glucoside (Isoquercitrin) | 0.005326 | 0.004602 | 0.005041 | 0.001888 | 0.002021 | 0.001732 | 0.38 | -1.41 | 1.31 | down |
| Quercetin-4'-O-glucoside (Spiraeoside) | 0.005623 | 0.004528 | 0.005006 | 0.001776 | 0.001760 | 0.001650 | 0.34 | -1.54 | 1.30 | down |
| Quercetin-4鈥?O-glucuronide | 0.000246 | 0.000467 | 0.000257 | 0.000066 | 0.000053 | 0.000068 | 0.19 | -2.32 | 1.16 | down |
| Quercetin-5-O-glucuronide | 0.000188 | 0.000380 | 0.000246 | 0.000039 | 0.000048 | 0.000060 | 0.18 | -2.43 | 1.17 | down |
| Quercetin-7-O-glucoside | 0.004798 | 0.003778 | 0.003785 | 0.001664 | 0.001279 | 0.001199 | 0.34 | -1.58 | 1.27 | down |
| Quercetin-7-O-rutinoside | 0.000198 | 0.000233 | 0.000167 | 0.000042 | 0.000024 | 0.000019 | 0.14 | -2.87 | 1.28 | down |
| Quercetin-7-O-rutinoside-4'-O-glucoside | 0.000138 | 0.000163 | 0.000178 | 0.000090 | 0.000028 | 0.000014 | 0.28 | -2.27 | 1.20 | down |
| Myricetin-3-O-galactoside | 0.000041 | 0.000046 | 0.000072 | 0.000017 | 0.000032 | 0.000023 | 0.45 | -1.14 | 1.07 | down |
| Phlorizin | 0.000085 | 0.000081 | 0.000056 | 0.000027 | 0.000013 | 0.000027 | 0.30 | -1.77 | 1.23 | down |

**Table S7.** Differential accumulation metabolites (DAMs) involved in stilbenoid and flavonoid pathways in MT2

| Metabolites | WT-1 | WT-2 | WT-3 | MT2-1 | MT2-2 | MT2-3 | Fold Change (FC) | log2FC | VIP | Regulated |
| --- | --- | --- | --- | --- | --- | --- | --- | --- | --- | --- |
| Resveratrol-3,5-di-O-glucoside | 0.000026 | 0.000015 | 0.000018 | 0.000433 | 0.000075 | 0.000176 | 11.56 | 3.22 | 1.08 | up |
| Piceatannol-3'-O-glucoside | 0.003933 | 0.001910 | 0.002983 | 0.014712 | 0.004911 | 0.010966 | 3.47 | 1.71 | 1.10 | up |
| Piceid | 0.000327 | 0.000229 | 0.000285 | 0.002440 | 0.000657 | 0.001355 | 5.30 | 2.22 | 1.06 | up |
| Cyanidin-3-O-(6''-O-caffeoyl)glucoside | 0.000261 | 0.000300 | 0.000149 | 0.000042 | 0.000032 | 0.000063 | 0.19 | -2.37 | 1.26 | down |
| Cyanidin-3-O-(6''-O-p-Coumaroyl)glucoside | 0.034795 | 0.034733 | 0.030649 | 0.009608 | 0.006300 | 0.006956 | 0.23 | -2.15 | 1.40 | down |
| Malvidin-3,5-di-O-glucoside (Malvin) | 0.001013 | 0.000837 | 0.001667 | 0.000299 | 0.000270 | 0.000255 | 0.23 | -2.03 | 1.24 | down |
| Malvidin-3-O-(6''-O-p-coumaroyl)glucoside | 0.000786 | 0.000785 | 0.001005 | 0.000062 | 0.000035 | 0.000039 | 0.05 | -4.29 | 1.40 | down |
| Malvidin-3-O-(6''-O-p-coumaroyl)glucoside-5-O-glucoside | 0.000498 | 0.000530 | 0.000724 | 0.000110 | 0.000086 | 0.000091 | 0.16 | -2.60 | 1.36 | down |
| Peonidin-3-O-(6''-O-Acetyl)glucoside | 0.000603 | 0.000417 | 0.000495 | 0.000077 | 0.000128 | 0.000191 | 0.26 | -2.02 | 1.33 | down |
| Peonidin-3-O-(6''-O-caffeoyl)glucoside | 0.000248 | 0.000266 | 0.000152 | 0.000058 | 0.000056 | 0.000042 | 0.23 | -2.07 | 1.30 | down |
| Peonidin-3-O-(6''-O-p-coumaroyl)glucoside | 0.036469 | 0.037956 | 0.035625 | 0.004170 | 0.003237 | 0.004947 | 0.11 | -3.18 | 1.41 | down |
| Peonidin-3-O-(6''-O-p-Coumaroylglucoside)-5-O-Glucoside | 0.004426 | 0.007164 | 0.008612 | 0.001606 | 0.002145 | 0.003404 | 0.35 | -1.51 | 1.21 | down |
| Peonidin-3-O-glucoside | 0.026342 | 0.024426 | 0.026136 | 0.006909 | 0.006530 | 0.008026 | 0.28 | -1.85 | 1.41 | down |
| Delphinidin-3-O-(6''-O-p-coumaroyl)glucoside | 0.000171 | 0.000217 | 0.000155 | 0.000060 | 0.000013 | 0.000020 | 0.17 | -2.85 | 1.35 | down |
| Pelargonidin-3-O-glucoside | 0.000459 | 0.000476 | 0.000426 | 0.000124 | 0.000140 | 0.000210 | 0.35 | -1.56 | 1.38 | down |
| Proanthocyanidins | 0.000068 | 0.000065 | 0.000061 | 0.000011 | 0.000006 | 0.000015 | 0.16 | -2.69 | 1.40 | down |
| Apigenin-7-O-(6''-p-Coumaryl)glucoside | 0.000596 | 0.000889 | 0.000638 | 0.000072 | 0.000065 | 0.000094 | 0.11 | -3.19 | 1.36 | down |
| Apigenin-7-O-glucoside(Cosmosiin) | 0.000008 | 0.000005 | 0.000016 | 0.000020 | 0.000018 | 0.000020 | 2.00 | 1.16 | 1.16 | up |
| Apigenin-7-O-neohesperidoside (Rhoifolin) | 0.000248 | 0.000363 | 0.000325 | 0.000032 | 0.000026 | 0.000042 | 0.11 | -3.23 | 1.38 | down |
| Apigenin-7-O-rutinoside (Isorhoifolin) | 0.000545 | 0.000813 | 0.000611 | 0.000069 | 0.000059 | 0.000107 | 0.12 | -3.09 | 1.36 | down |
| Dihydrokaempferol-3-O-glucoside | 0.000761 | 0.000724 | 0.000803 | 0.002805 | 0.001626 | 0.002114 | 2.86 | 1.48 | 1.27 | up |
| Dihydrokaempferol-7-O-glucoside | 0.000234 | 0.000198 | 0.000192 | 0.001227 | 0.000784 | 0.001036 | 4.89 | 2.27 | 1.34 | up |
| Eriodictyol (5,7,3',4'-Tetrahydroxyflavanone) | 0.000005 | 0.000010 | 0.000005 | 0.000043 | 0.000043 | 0.000037 | 5.93 | 2.65 | 1.40 | up |
| Eriodictyol 7-O-β-D-glucopyranoside | 0.000799 | 0.000758 | 0.000797 | 0.002794 | 0.001630 | 0.001895 | 2.69 | 1.39 | 1.24 | up |
| Kaempferol-3,7-di-O-glucoside | 0.000282 | 0.000319 | 0.000200 | 0.000051 | 0.000017 | 0.000052 | 0.15 | -2.89 | 1.34 | down |
| Kaempferol-3-caffeoyldiglucoside | 0.000024 | 0.000032 | 0.000043 | 0.000007 | 0.000019 | 0.000012 | 0.39 | -1.41 | 1.19 | down |
| Kaempferol-3-O-(6''-p-Coumaroyl)glucoside (Tiliroside) | 0.000040 | 0.000052 | 0.000029 | 0.000006 | 0.000005 | 0.000010 | 0.17 | -2.58 | 1.31 | down |
| Kaempferol-3-O-glucorhamnoside | 0.033654 | 0.035255 | 0.031021 | 0.008763 | 0.006590 | 0.007231 | 0.23 | -2.15 | 1.41 | down |
| Kaempferol-3-O-neohesperidoside | 0.038414 | 0.041159 | 0.034743 | 0.010572 | 0.007612 | 0.006725 | 0.22 | -2.22 | 1.40 | down |
| Kaempferol-3-O-rutinoside(Nicotiflorin) | 0.034121 | 0.036454 | 0.029662 | 0.009660 | 0.006721 | 0.008059 | 0.24 | -2.05 | 1.39 | down |
| Kaempferol-3-O-rutinoside-7-O-glucoside | 0.001104 | 0.001344 | 0.001946 | 0.000356 | 0.000700 | 0.000890 | 0.44 | -1.23 | 1.15 | down |
| Luteolin-4'-O-glucoside | 0.000095 | 0.000166 | 0.000177 | 0.000050 | 0.000037 | 0.000030 | 0.27 | -1.88 | 1.28 | down |
| Luteolin-7,3'-di-O-glucoside | 0.000297 | 0.000331 | 0.000201 | 0.000076 | 0.000043 | 0.000102 | 0.27 | -1.96 | 1.30 | down |
| Luteolin-7-O-(6''-caffeoyl)rhamnoside | 0.000041 | 0.000050 | 0.000031 | 0.000008 | 0.000005 | 0.000008 | 0.17 | -2.54 | 1.34 | down |
| Luteolin-7-O-gentiobioside | 0.000300 | 0.000273 | 0.000184 | 0.000099 | 0.000022 | 0.000059 | 0.24 | -2.30 | 1.29 | down |
| Luteolin-7-O-glucoside (Cynaroside) | 0.000342 | 0.000273 | 0.000161 | 0.000450 | 0.001134 | 0.000851 | 3.14 | 1.62 | 1.15 | up |
| Naringenin (5,7,4'-Trihydroxyflavanone) | 0.000009 | 0.000016 | 0.000008 | 0.000087 | 0.000103 | 0.000091 | 8.28 | 3.11 | 1.40 | up |
| Naringenin-7-O-glucoside (Prunin) | 0.001434 | 0.001529 | 0.001355 | 0.003492 | 0.004434 | 0.004023 | 2.77 | 1.46 | 1.39 | up |
| Quercetin-3-O-(2'''-Caffeoyl)sophoroside | 0.000004 | 0.000008 | 0.000008 | 0.000000 | 0.000000 | 0.000000 | 0.00 | -10.24 | 1.31 | down |
| Quercetin-3-O-(2''-O-glucosyl)glucuronide | 0.000284 | 0.000519 | 0.000403 | 0.000015 | 0.000013 | 0.000010 | 0.03 | -4.95 | 1.34 | down |
| Quercetin-3-O-(6''-p-Coumaroyl)galactoside | 0.001405 | 0.001628 | 0.000951 | 0.000126 | 0.000063 | 0.000078 | 0.07 | -3.92 | 1.34 | down |
| Quercetin-4-O-glucuronide | 0.000246 | 0.000467 | 0.000257 | 0.000027 | 0.000018 | 0.000009 | 0.06 | -4.23 | 1.28 | down |
| Quercetin-5-O-glucuronide | 0.000188 | 0.000380 | 0.000246 | 0.000022 | 0.000014 | 0.000010 | 0.06 | -4.15 | 1.30 | down |
| Quercetin-7-O-rutinoside | 0.000198 | 0.000233 | 0.000167 | 0.000070 | 0.000023 | 0.000040 | 0.22 | -2.30 | 1.35 | down |
| Quercetin-7-O-rutinoside-4'-O-glucoside | 0.000138 | 0.000163 | 0.000178 | 0.000041 | 0.000042 | 0.000071 | 0.32 | -1.67 | 1.36 | down |
| Pinocembrin-7-O-glucoside (Pinocembroside) | 0.000006 | 0.000004 | 0.000004 | 0.000064 | 0.000072 | 0.000142 | 19.90 | 4.24 | 1.24 | up |
| Protocatechuic acid-4-O-glucoside | 0.000478 | 0.000644 | 0.000707 | 0.000254 | 0.000173 | 0.000200 | 0.34 | -1.55 | 1.34 | down |

**Table S8.** Statistics of RNA-seq data quality

| Samples | Clean reads | Clean bases | GC content (%) | Q20(%) | Q30(%) |
| --- | --- | --- | --- | --- | --- |
| WT-1 | 22726912 | 6779975590 | 46.76 | 98.36 | 94.96 |
| WT-2 | 21501274 | 6405338144 | 46.6 | 98.26 | 94.78 |
| WT-3 | 20767758 | 6188585360 | 46.66 | 98.3 | 94.91 |
| MT1-1 | 27214786 | 8109266184 | 46.76 | 98.2 | 94.63 |
| MT1-2 | 24480850 | 7298342402 | 46.99 | 98.31 | 94.93 |
| MT1-3 | 25104081 | 7487681272 | 46.59 | 98.32 | 94.92 |
| MT2-1 | 28970147 | 8635403272 | 47.12 | 98.23 | 94.72 |
| MT2-2 | 22215717 | 6629903396 | 47.22 | 98.11 | 94.45 |
| MT2-3 | 22059590 | 6596375138 | 46.72 | 98.25 | 94.75 |

**Table S9.** Statistics on data mapping

| Sample | Total Reads | Mapped Reads | Uniq Mapped Reads | Multiple Map Reads | Reads Map to '+' | Reads Map to '-' |
| --- | --- | --- | --- | --- | --- | --- |
| WT-1 | 45,453,824 | 41,499,609 (91.30%) | 39,951,948 (87.90%) | 1,547,661 (3.40%) | 21,993,420 (48.39%) | 21,895,477 (48.17%) |
| WT-2 | 43,002,548 | 39,004,831 (90.70%) | 37,473,119 (87.14%) | 1,531,712 (3.56%) | 20,710,993 (48.16%) | 20,698,874 (48.13%) |
| WT-3 | 41,535,516 | 37,805,974 (91.02%) | 36,394,498 (87.62%) | 1,411,476 (3.40%) | 20,011,951 (48.18%) | 19,990,917 (48.13%) |
| MT1-1 | 54,429,572 | 49,774,544 (91.45%) | 47,217,661 (86.75%) | 2,556,883 (4.70%) | 27,150,410 (49.88%) | 27,152,734 (49.89%) |
| MT1-2 | 48,961,700 | 44,820,361 (91.54%) | 42,131,402 (86.05%) | 2,688,959 (5.49%) | 24,929,512 (50.92%) | 24,895,270 (50.85%) |
| MT1-3 | 50,208,162 | 46,088,288 (91.79%) | 43,708,733 (87.06%) | 2,379,555 (4.74%) | 25,221,762 (50.23%) | 25,201,696 (50.19%) |
| MT2-1 | 57,940,294 | 52,765,802 (91.07%) | 48,626,916 (83.93%) | 4,138,886 (7.14%) | 30,547,647 (52.72%) | 30,595,997 (52.81%) |
| MT2-2 | 44,431,434 | 40,333,607 (90.78%) | 37,924,733 (85.36%) | 2,408,874 (5.42%) | 22,407,769 (50.43%) | 22,386,054 (50.38%) |
| MT2-3 | 44,119,180 | 40,054,233 (90.79%) | 37,625,798 (85.28%) | 2,428,435 (5.50%) | 22,277,120 (50.49%) | 22,261,562 (50.46%) |

Note:

Total Reads: Counts of Clean Reads, counted as single end;

Mapped Reads: Counts of mapped reads and the proportion of that in clean data;

Uniq Mapped Reads: Counts of reads mapped to a unique position on reference genome and proportion of that in clean data;

Multiple Mapped Reads: Counts of reads mapped to multiple positions on reference genome and proportion of that in clean data;

Reads Map to '+': Counts of reads mapped to the sense chain and the proportion of that in clean data;

Reads Map to '-': Counts of reads mapped to antisense chain and proportion of that in clean data.


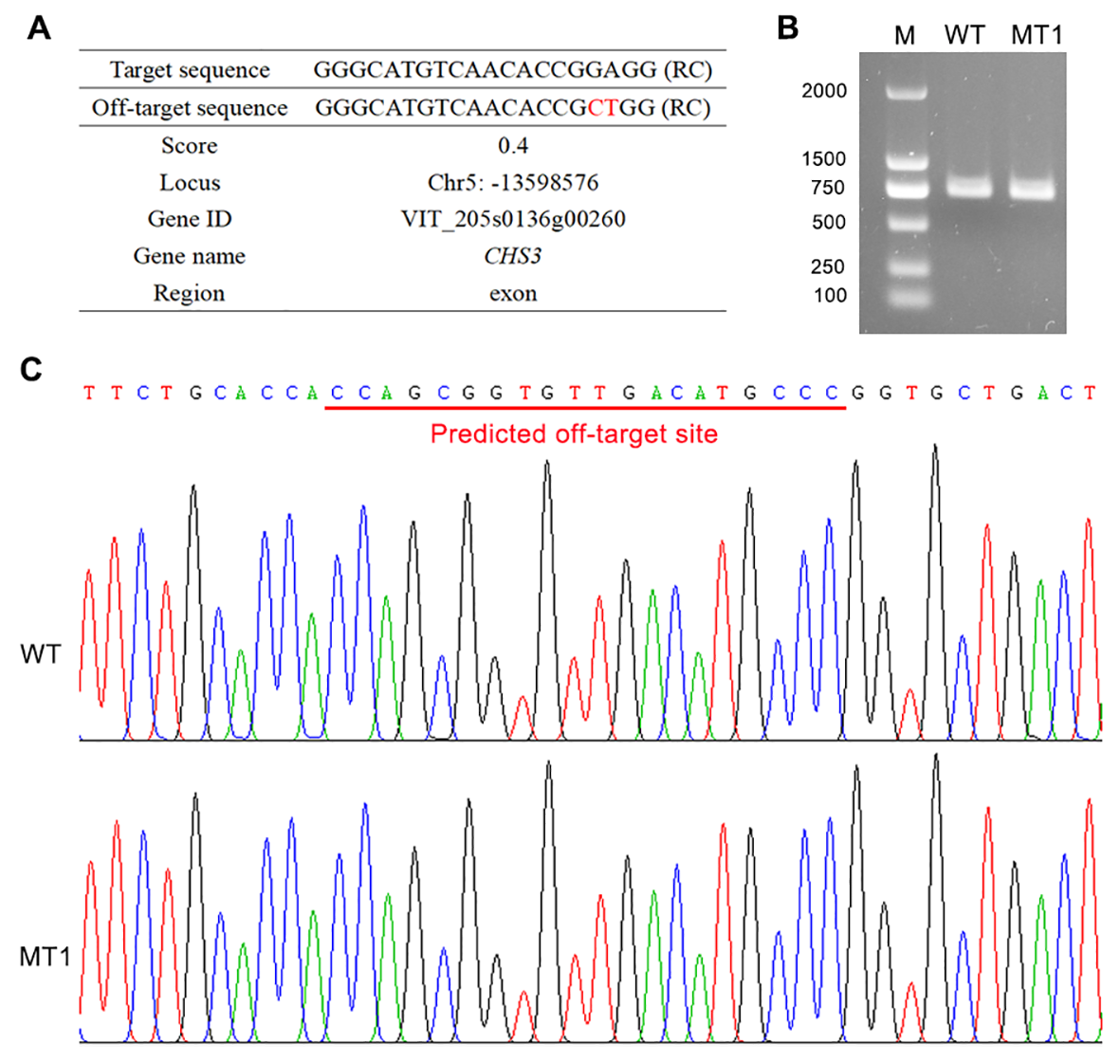


**Figure S1.** Prediction and identification of off-target

(A) Prediction of off-target. (B) PCR amplification of off-target in *CHS3*.

(C) Sequencing chromatograms of predicted off-target site.


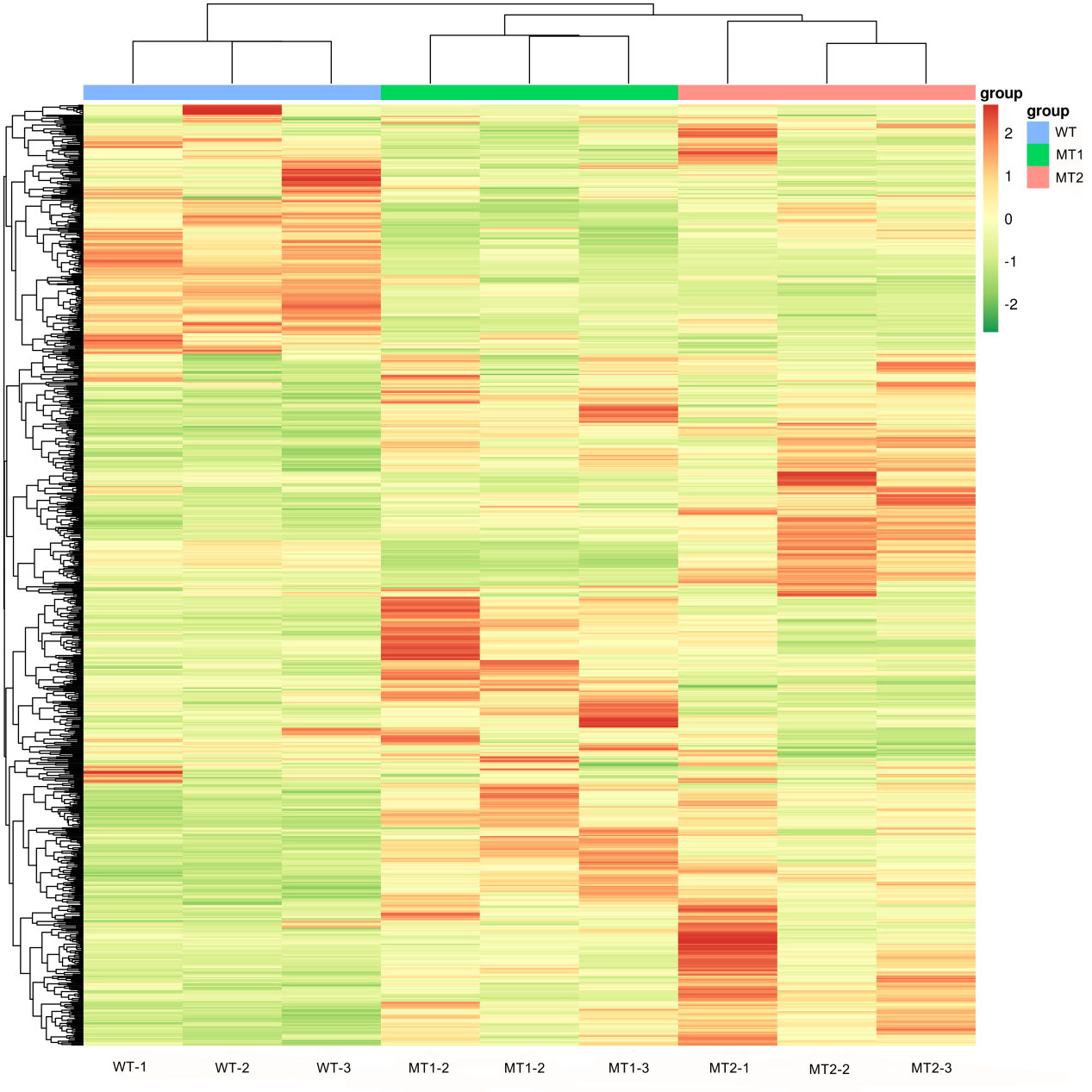


**Figure S2.** Clustering heat map of metabolite accumulation in different samples


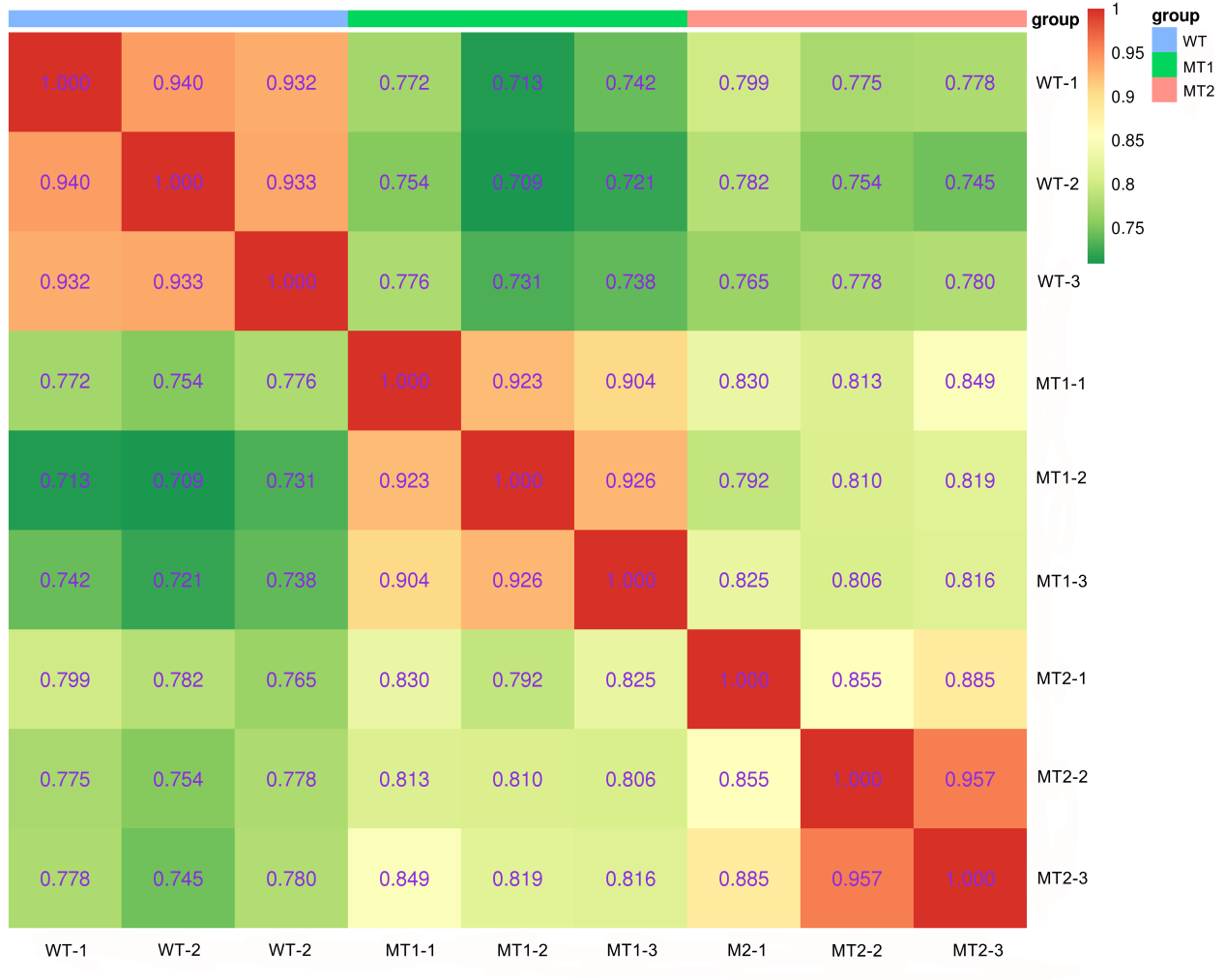


**Figure S3.** Correlation analysis of metabolite accumulation in different sample


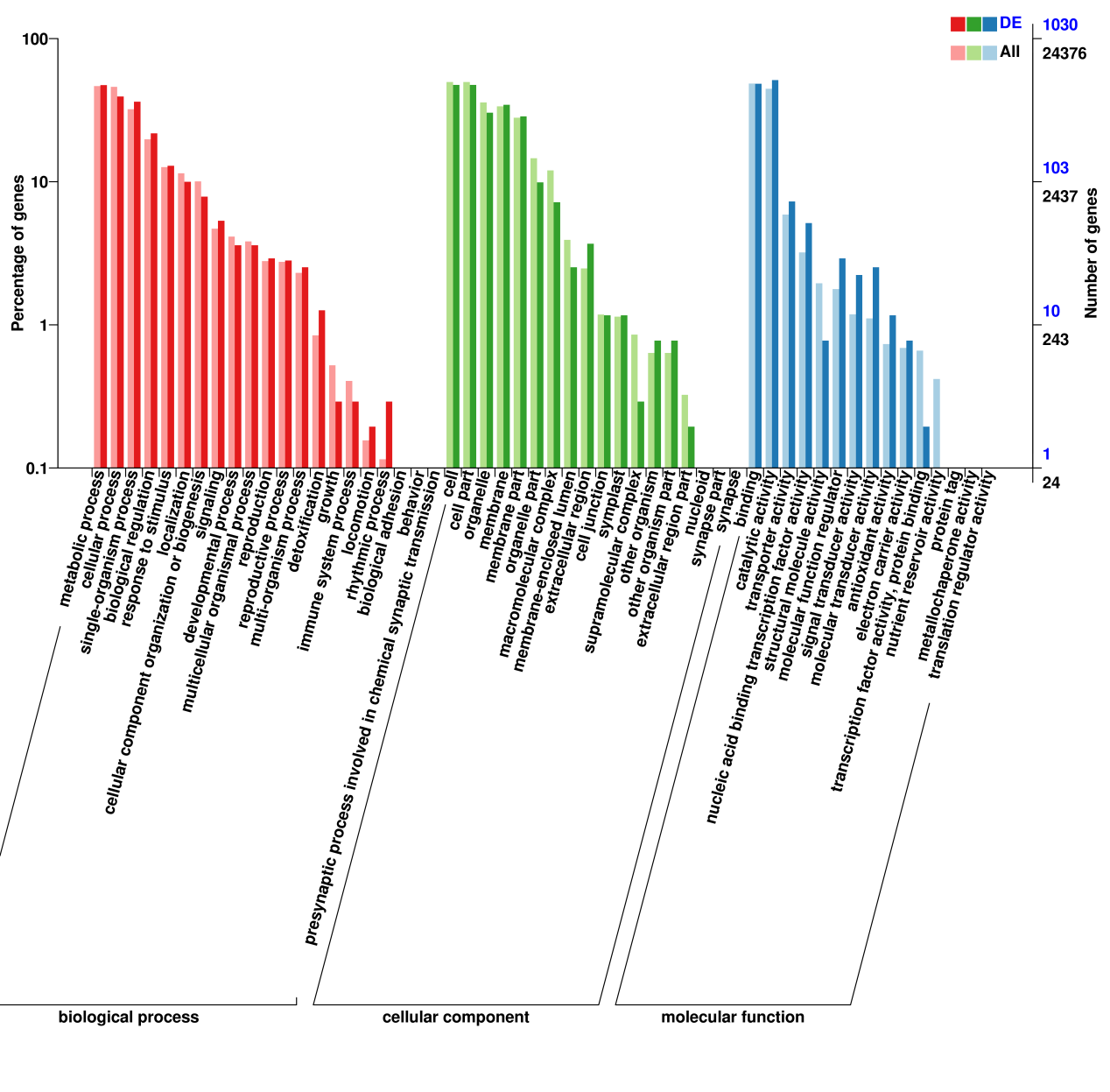


**Figure S4.** The classification of Gene Ontology (GO) annotation in MT1


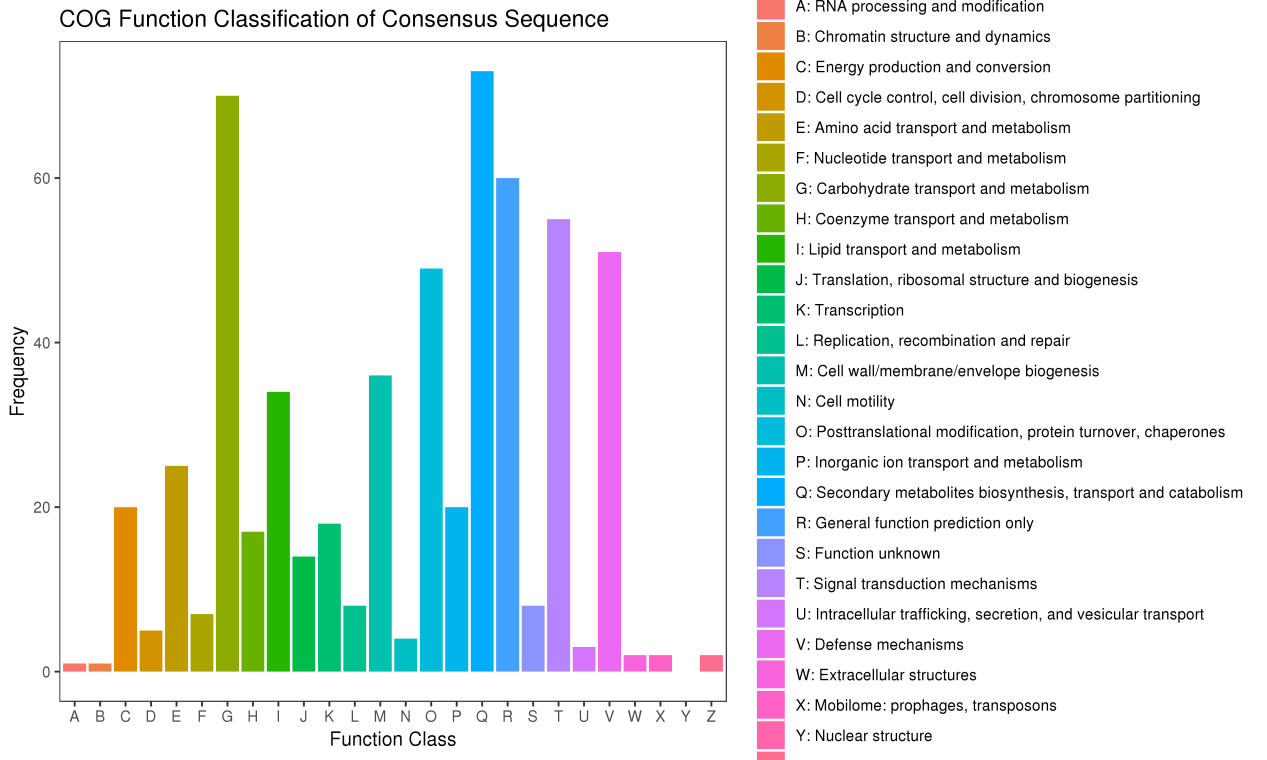


**Figure S5.** The classification of the clusters of orthologous groups (COG) annotation in MT1


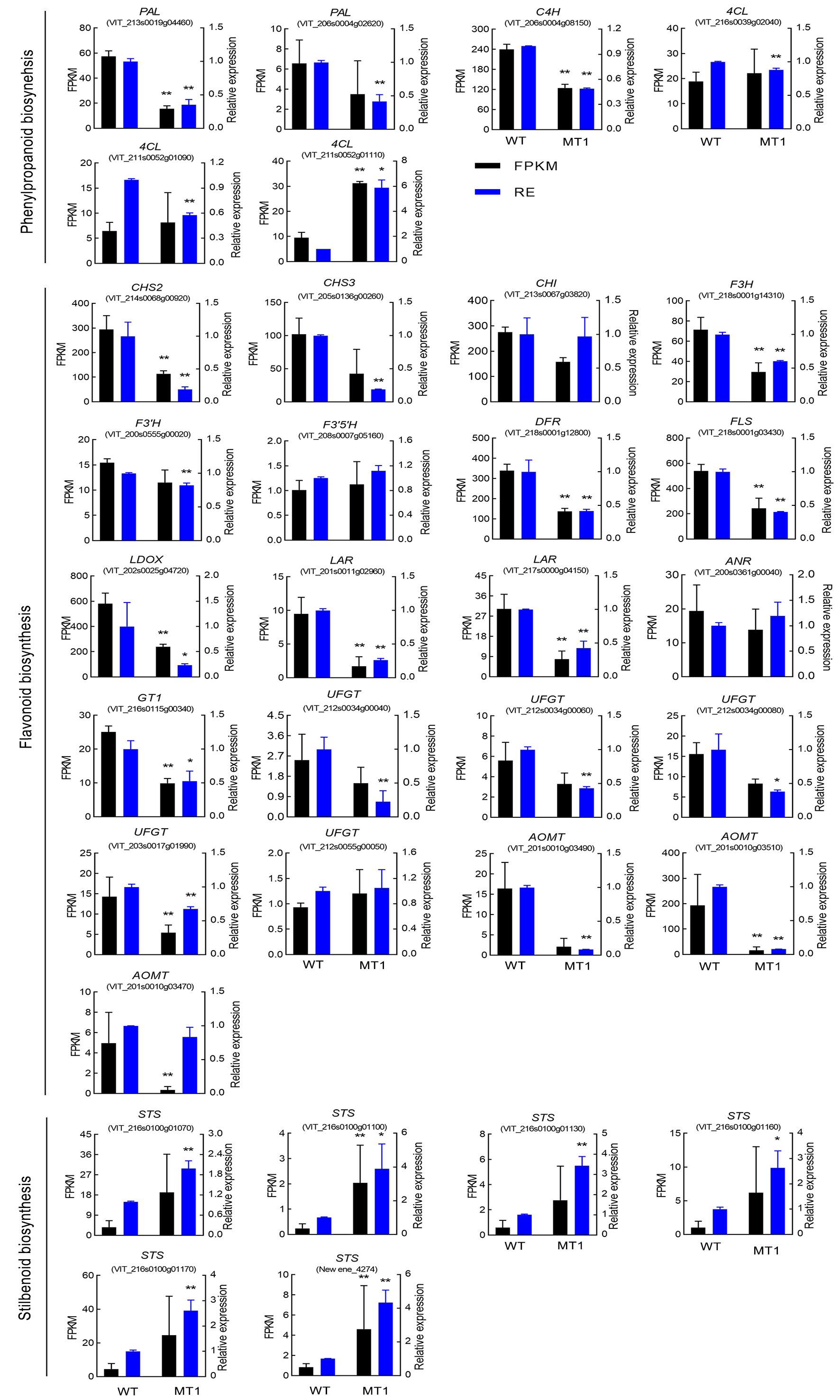


**Figure S6.** The gene relative expression patterns of mutation cell lines involved in phenylpropanoid, flavonoid and stilbenoid biosynthesis

Relative expression of 33 genes were detected involved in phenylpropanoid, flavonoid and stilbenoid biosynthesis. * and ** represent significant differences at the 0.05 and 0.01 level between WT and MT1 mutation cell line.
